# Structural snapshots of human pre-60S ribosomal particles before and after nuclear export

**DOI:** 10.1038/s41467-020-17237-x

**Published:** 2020-07-15

**Authors:** Xiaomeng Liang, Mei-Qing Zuo, Yunyang Zhang, Ningning Li, Chengying Ma, Meng-Qiu Dong, Ning Gao

**Affiliations:** 1 State Key Laboratory of Membrane Biology, School of Life Science, Tsinghua University, 100084 Beijing, China; 2 State Key Laboratory of Membrane Biology, Peking-Tsinghua Joint Centre for Life Sciences, School of Life Sciences, Peking University, 100871 Beijing, China; 3 College of Biological Sciences, China Agricultural University, 100193 Beijing, China; 4 National Institute of Biological Sciences, 102206 Beijing, China; 5 Tsinghua Institute of Multidisciplinary Biomedical Research, Tsinghua University, 100084 Beijing, China

**Keywords:** Cryoelectron microscopy, Ribosome

## Abstract

Ribosome biogenesis is an elaborate and energetically expensive program that involve two hundred protein factors in eukaryotes. Nuclear export of pre-ribosomal particles is one central step which also serves as an internal structural checkpoint to ensure the proper completion of nuclear assembly events. Here we present four structures of human pre-60S particles isolated through a nuclear export factor NMD3, representing assembly stages immediately before and after nuclear export. These structures reveal locations of a dozen of human factors, including an uncharacterized factor TMA16 localized between the 5S RNA and the P0 stalk. Comparison of these structures shows a progressive maturation for the functional regions, such as peptidyl transferase centre and peptide exit tunnel, and illustrate a sequence of factor-assisted rRNA maturation events. These data facilitate our understanding of the global conservation of ribosome assembly in eukaryotes and species-specific features of human assembly factors.

## Abstract (web summary)

Ribosome biogenesis in eukaryotes is a complex process that involves more than 200 protein factors. Here the authors present a structural analysis of a collection of human pre-60S structures sampled through a nuclear export adaptor NMD3, representing structural snapshots of pre-60S particles immediately before and after passing through nuclear pore complex.

## Introduction

Our knowledge on eukaryotic ribosome biogenesis is mainly from several decades of genetic and biochemical studies using the model organism of *Saccharomyces cerevisiae*^1–4^. The assembly of ribosomal subunits starts co-transcriptionally in nucleolus on growing nascent rRNA transcripts, followed by continuous maturation in nucleoplasm and is completed in cytoplasm. After an early endonucleolytic cleavage that generates rRNA precursors for the large and small subunits, further assembly of the two subunits takes separate routes, and each is coupled with additional enzymatic processing and modification of rRNA precursors. In general, assembly factors, organized in temporal and spatial groups, facilitate the conformational maturation of phylogenetically conserved structural domains of rRNA in a progressive manner, and some of them have a regulatory role in surveillance and quality control of the assembly process^2–6^. Importantly, there are extensive cross-talks between ribosome biogenesis and other cellular processes, and dysfunction of ribosome biogenesis has been linked to cancer initiation and progression in human^7–9^. In addition, a group of diverse human genetic disorders, termed ribosomopathies^10,11^, is directly linked to mutations on ribosomal proteins and assembly factors. The clinical phenotypes of these diseases are heterogenous, but patients with these mutations share a predisposition to a variety of cancers^9,12^.

In recent years, our understanding of the general pictures of the assembly process and the diverse functions of assembly factors has been greatly advanced by cryo-electron microscopy (cryo-EM) structures of *fungi* pre-ribosomal particles at different assembly stages [reviewed in ref. ^2,4^]. Although ribosome biogenesis is considered generally conserved in eukaryotes, it is currently unclear to what extent or which step of ribosome biogenesis differs among species. Human ribosome assembly is less studied, and only very few putative human homologs of known yeast factors have been examined. In addition, human cells possess many additional rRNA processing factors that share no homology with yeast factors, and therefore may have distinct assembly and regulation pathways^13–16^. Structural characterization of assembly intermediates could be an efficient way to expand our knowledge in the human ribosome biosynthesis. Previously, structures of human pre-ribosomal precursors have been reported for pre-40S particles in late cytoplasmic stages^17,18^, however, such information for human pre-60S particles is still lacking.

Here we report the cryo-EM structures of human pre-60S particles in four sequential assembly states, which provide numerous details for the general principles and human-specific mechanisms of ribosome assembly in stages immediately before and after nuclear export.

## Results and discussion

### Structures of pre-60S particles purified through NMD3

**Fig. 1:**
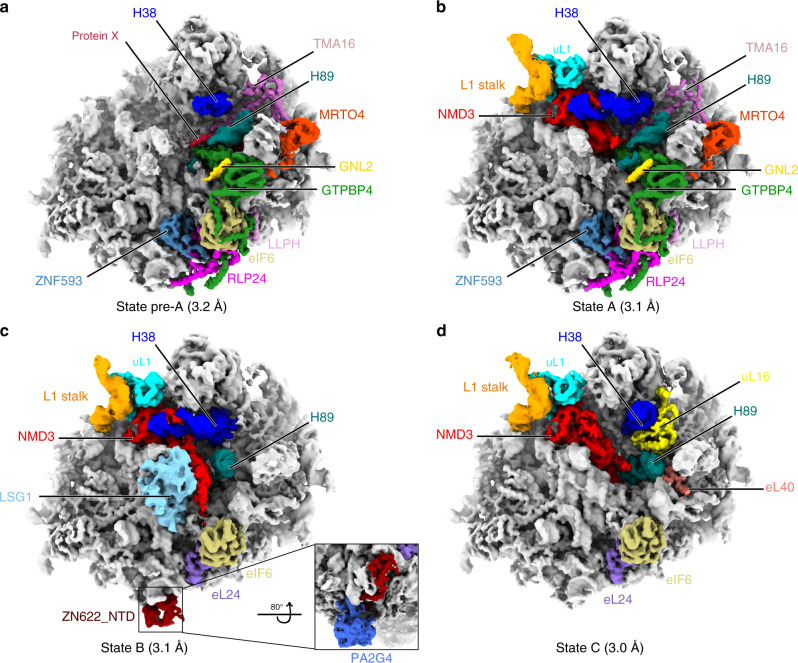
Structures of human pre-60S particles isolated through C-terminally tagged NMD3. **a**–**d**, Cryo-EM maps of the four structures of NMD3-particles from pre-A to C, gaussian filtered with sDev of 1.058 Å in ChimeraX^70^. Individual assembly factors, H38, H89, L1 stalk, eL24, eL40 and uL1 are color-coded. Below each map, the overall resolution is shown.

To obtain native pre-60S particles from human cells, we first generated a HEK293 cell line stably expressing a pre-60S nuclear export factor NMD3^19,20^ with an affinity tag at the C-terminus (NMD3-tev-3XFLAG). Following affinity purification (Supplementary Fig. 1a), pre-60S particles were characterized using cryo-EM. Through global and focused 3D classification, we obtained a set of pre-60S structures (states pre-A, A-C, resolution 3.0–3.2 Å) (Supplementary Figs. 1d, 2, Supplementary Table 1), representing four consecutive late-nucleoplasmic to mid-cytoplasmic assembly stages that span the lifetime of NMD3 on the pre-60S particles (Fig. 1, Supplementary Movie 1). Among these states, state A represents a stage right before nuclear export, as the rotation of the 5S RNP has already occurred. State A is with stable binding of NMD3, GTPBP4 (Nog1 in yeast), MROT4, eIF6, RLP24, LLPH (YBL028C in yeast), ZNF593 (Bud20 in yeast) and a previously uncharacterized factor TMA16 (Fig. 1b, Supplementary Fig. 3). Interestingly, a short helix (residues 160–178) of GNL2 (Nog2 in yeast) is also visible at its conserved binding site on the surface of GTPBP4, indicating that state A is a nucleoplasmic intermediate after the stable association of NMD3 but before the complete departure of the nuclear factor GNL2^21^. In contrast, in state pre-A NMD3 is not fully accommodated: L1 stalk is highly flexible (Fig. 1a), and smeared densities of NMD3 are only visible at very low threshold. Another major difference of state pre-A is that a piece of rod-like density, a ~28-residue α-helix (Supplementary Fig. 4a–c), is present at the peptidyl transferase center (PTC). This α-helix is sufficiently resolved to determine its polarity (Supplementary Fig. 4d). The C-terminal end of this unidentified protein (protein X) has a steric clash with the N-terminal domain (NTD) of NMD3. Tracing of its N-terminal part shows that it sits underneath H89, passes between H42 and H91, and exposes it N-terminal end to the solvent in a cavity of H91, H97 and uL6 (Supplementary Fig. 4e–f, Supplementary Movie 2). Unfortunately, through multiple rounds of crosslinking coupled with mass-spectrometry (CXMS) and extensive efforts in trial-and-error modeling of candidate sequences, we had no success in revealing its identity. Nevertheless, its conflict with NMD3-NTD suggests that the release of this factor might be a prerequisite for the stable binding of NMD3. In addition, the presence of MRTO4 in state pre-A and A (Fig. 1a,b) also confirms that they are nucleoplasmic stages, as the release of MRTO4 is coupled with the recruitment of export factors to the P0 stalk^22^.

In state B, the cytoplasmic release of GTPBP4, RLP24 and ZNF593 has taken place and LSG1 sits on the top of H69, making contacts with the NTD of NMD3 (Fig. 1c), similar to the yeast pre-60S particles at similar stages^23,24^. Concomitant to the factor changes at the PTC, ZNF622 (Rei1 in yeast) and PA2G4 (Arx1 in yeast) bind at the peptide tunnel exit (PTE) of state B. In the following state C, LSG1 is released, and the PTC is in a nearly mature conformation with the incorporation of uL16 (Fig. 1d). In both states B and C, N-terminal region of ZNF622 and PA2G4 are not in high-resolution. Focused classification on the region of ZNF622 and PA2G4 could provide positive identification of these two factors and allow the docking of the atomic models of PA2G4 (PDB: 6SXO and 2Q8K)^25,26^ and a homology model of ZNF622.

In terms of ribosomal proteins, gradual incorporation of several late-binding proteins could be seen in these states. eL41 is missing from all the states, whereas eL40 and uL16 start to appear in state C (Fig. 1d). In addition, replacement of RLP24 with eL24 takes place as the pre-60S particles transit from state A to B (Fig. 1b,c). In agreement with yeast pre-60S structures^23,24^, uL16 binding is coupled with the repositioning of H38 in its near mature conformation. The flexible P0 stalk is not well resolved in all the states, but mass-spectrometry data shows that in addition to MRTO4, uL11, P0, P1, P2, and DUSP12 (Yvh1 in yeast) are present in the sample (Supplementary Table 2), suggesting that the stalk in also in progressive maturation stages.

### TMA16 is an uncharacterized nuclear assembly factor

**Fig. 2:**
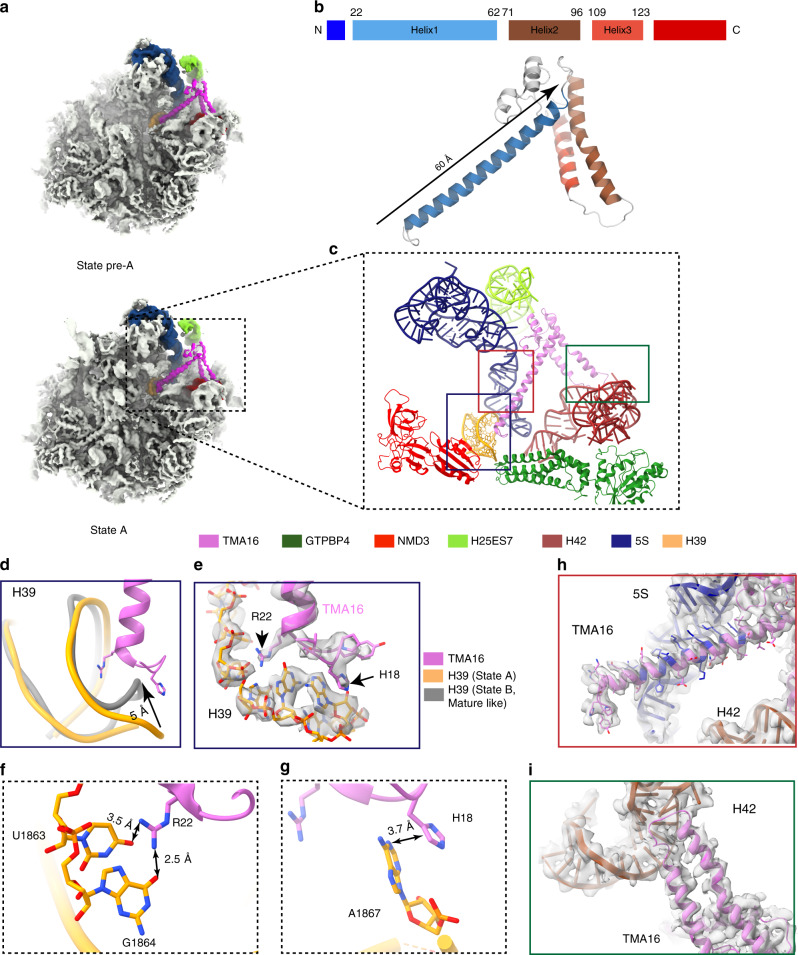
Structure of human TMA16. **a** Cryo-EM maps of states pre-A and state A (Gaussian filtered with sDev of 1.058 Å), with TMA16, 5S rRNA, part of H25ES7, H39 and H42 color-coded. **b** Secondary structural elements and the atomic model of human TMA16. **c** Zoom-in view showing the surrounding of TMA16. Two PTC-located assembly factors are also shown (NMD3 and GTPBP4). The three boxed regions are further detailed in panels **d**, **g** and **h**. **d** Comparison of H39 in state A (orange) and state B (gray). **e** Interactions between the N-terminus of TMA16 and H39 (immature conformation) in state A. **f** R22 of TMA16 interacts with G1864 of H39. **g**, H18 of TMA16 stacks with the base of A1867 of H39. **h**, Interactions between the helix 1 of TMA16 and the 5S rRNA in state A. **i**, Interactions between the H2-H3 loop region of TMA16 and H42 of the 28S rRNA.

In the maps of states pre-A and A, a previously uncharacterized factor is seen to bind in the space between the rotated 5S RNP and the P0 stalk (Fig. 2a). Facilitated by CXMS, which reported a crosslink between K114-TMA16 and K136- RPL18A (eL20), the factor was identified to be human TMA16 (Supplementary Fig. 5a,b). An atomic model could be built for residues 17–166 (full-length 203 residues). In the N-terminus, a long α-helix (residue 19–60, ~60 Å) points to the PTC, with its N-terminal end docks onto the tip of H39 of the 28S rRNA (Fig. 2b,c). Two conserved residues of TMA16, H18 and R22, directly interacts with A1867 and G1864 of H39, respectively (Fig. 2d–f). The tip of H39 in state A is seen to have a distortion (up to 5 Å) to accommodate TMA16 (Fig. 2d). In states B and C, H39 adopts a mature conformation in the absence of TMA16. In addition, this N-terminal helix also has extensive interactions with the 5S RNA (Fig. 2h), mainly through its basic residues. The main body of TMA16 is sandwiched between H42 and H25ES7 (Supplementary Fig. 6a), with one side (the loop between H1 and H2 and the C-terminal region of TMA16) interacting with H25ES7, and the other side (the loop between H2 and H3) specifically contacting H42 (Fig. 2g). This strategic position of TMA16 suggests that it may sense the rotation of the 5S RNP and its binding to the empty space between H42 and H25ES7 could be important in stabilizing the pre-60S particles during nuclear export, underlining the possibility that TMA16 is another nuclear export adapter.

TMA16 is conserved in eukaryotes (Supplementary Fig. 6g), and it was reported to have physical or genetic interaction with several ribosome biogenesis/export factors, including Crm1/XPO1, in yeast or human cells from previous proteomics studies^27–30^. Very recently, yeast Tma16 was found to be a component of pre-60S particles purified through Arx1 or Lsg1^31^. However, equivalent positions in the yeast pre-60S structures at similar stages are empty (Supplementary Fig. 6b). This suggests that TMA16 might be a transient binding factor, and the timing of its association and departure is strictly controlled. This position of TMA16 on the pre-60S particles is incompatible with several factors that act earlier than NMD3 (Supplementary Fig. 6c,d), including Nug1 and Cgr1 identified in the nucleoplasmic Nog2-particles^32^. It also has a steric clash with uL16. A close examination of the TMA16-H39 interface shows that GTPBP4-NTD has a direct role in stabilizing this interface: An aromatic residue Y124 of GTPBP4 is able to enhance the stacking pair between H18 of TMA16 and A1867 of H39 (Supplementary Fig. 6e,f). These observations suggest a relatively short time window for TMA16 action: after the 5S RNP rotation and before the departure of GTPBP4. This hypothesis is supported by recent findings that yeast Tma16 accumulates on the pre-60S Lsg1-particles when the release of Nog1 is impaired by mutations on Nog1 or Drg1^31^.

### Structures of conserved human pre-60S assembly factors

The factors identified in these pre-60S structures are evidently highly conserved in function, as their structures closely resemble their counterparts in yeast^23,24,32–34^. But the sequence conservation of some factors, such as LLPH, is limited, and identification of LLPH in the map relied largely on side-chain density matching. Among these factors, NMD3, TMA16, RLP24, ZNF593, eIF6 and GTPBP4 are well resolved, which enabled atomic modeling for the majority (or nearly entire) of their sequences (Supplementary Figs. 2 and 3). These factors, located in the equivalent positions as seen in the yeast pre-60S particles, interact with their partners in a highly conserved fashion. Such an example is GTPBP4, which is an organization hub interacting with multiple distant ribosomal proteins, assembly factors and rRNA helices along its path from the GTPase domain to the long C-terminal extension (Fig. 1a,b).

**Fig. 3:**
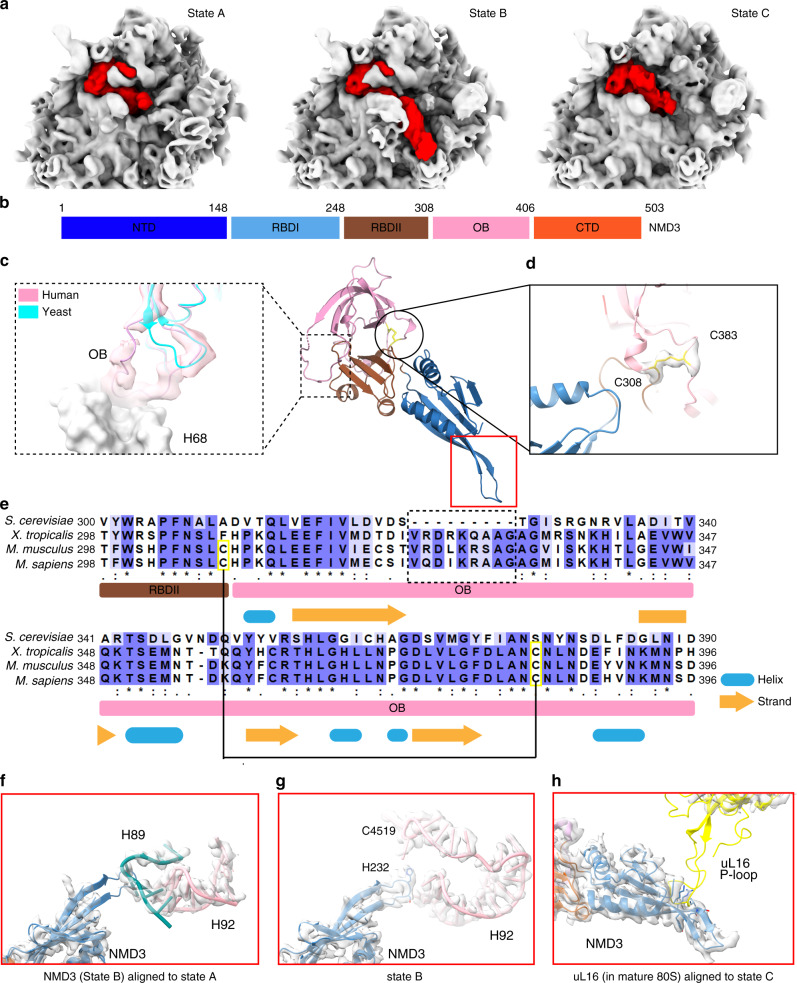
Structural dynamics of NMD3 in human pre-60S particles. **a**, Conformational changes of NMD3 on its N-terminal domain in the map of state A, B and C. **b** Domain organization of human NMD3. **c** Superimposition of one loop at the OB domain between the human (red) and yeast (cyan) NMD3 (PDB 5H4P)^33^. Domains of human NMD3 are separately colored. The segmented density map of NMD3 in state A is also shown. The lengthened loop in human NMD3 is capable of interacting with H68 of the 28S rRNA. **d** Zoom-in view on a mammal-specific disulfide bond in human NMD3 (yellow). Local density of the disulphide bond is also shown. **e** Sequence alignment of NMD3. The lengthened loop is labeled by a dash-lined square and residues of disulfide bond are highlighted in yellow. **f** Superimposition of the β4-β5 loop from NMD3 (state B) with the immature H89-H92 (state A), showing that this PTC-interacting motif clashes with the immature H89. **g** Interaction of the β4-β5 loop with the mature-like PTC components (H92 and C4519 of H90 in state B. **h** Superimposition of uL16 from the 80S ribosome (PDB 6EK0)^62^ with NMD3 (state C), indicating a conflict between the P-loop of uL16 and the β4-β5 loop of NMD3.

There are also a few species-specific features for some of these assembly factors. NMD3 could be clear seen in states A to C, and similar to yeast Nmd3^23,24,33,34^, it displays different conformations, mainly on its NTD (Fig. 3a,b). Human NMD3 possesses a long loop at the C-terminal OB domain (Fig. 3c), which is capable of interacting with H68. In contrast, the equivalent loop of yeast NMD3 is 10-residue shorter (Fig. 3e), and devoid of rRNA contact. Furthermore, a specific disulfide bond was seen between C308 and C383 in human NMD3, likely to provide extra stabilization for the OB domain (Fig. 3d).

For another example, compared with yeast Bud20, human ZNF593 contains a highly positively charged N-terminal tail, which is deeply embedded in a cavity surrounded by H35, H61, H62, H64 and H96 (Supplementary Fig. 7a–e). These helices are from different parts of the rRNA, including domain 0, II, IV and VI. Therefore, this tail might have a role in stabilizing the interface between different rRNA domains. This idea is in consistent with current model^2^ that Bud20 assembles in late nucleolar stages^35^ when more regions of Domains IV and V become ordered (Supplementary Fig. 7f–g) [the transition from State E (No Bud20)^36^ to the Nog2-particle (with Bud20)^32^]. The sequence difference between yeast and human might suggest that the folding kinetics of this multiple-domain interface is different among species.

As to ZNF622, its C-terminal tunnel-probing portion is well resolved but the N-terminal domain is less ordered. As observed in the yeast structure^37^, the NTD interacts with ES41, Helix 59 and eL22, resulting in a change on ES41 that would be incompatible with the formation of mature 80S ribosome by clashing with eS8 of the 40S subunit (Supplementary Fig. 8a,b). In addition, a human-specific interaction between ZNF622-NTD and ES27B could be found (Supplementary Fig. 8c,d). This is likely due to the fact that ES27 is much longer in the human ribosome.

PA2G4 (or EBP1) is the most diverged factor among the factors analyzed in our structures. PA2G4 has 394 residues in length, significantly shorter than its yeast homolog Arx1 (593 residues). It has two major splicing isoforms (isoform 1; isoform 2 lacking N-terminal 54 residues), and was mostly studied as a signaling molecule in the context of cancer. The two isoforms were reported to have opposing functions with regard to cell proliferation and differentiation^38^. The implication of PA2G4 in ribosome assembly was based on its presence in rRNA-containing complexes^39^. However, its exact molecular role is not settled and two very recent structures, one on the in vitro recombinant 80S-PA2G4 complex^25^ and the other on the ex vivo 80S-PA2G4 complex^40^ have emphasized an additional role in co-translational regulation. In our structures, the general position of PA2G4 is in a conserved site involving tunnel exit components, ES27, H59, eL19 and uL23 (Supplementary Fig. 9a–f). But it no longer has significant contacts with uL29, uL24 and the 5.8S rRNA (Supplementary Fig. 9g–k) as seen in the yeast structures^32,37,41,42^. Compared with the 80S-PA2G4 complex^25^, PA2G4 in the pre-60S structure is seen to have a 5-Å shift away from pre-60S body (Supplementary Fig. 9l–n). It is currently unclear to what extent these reported diverse functions of PA2G4 could be directly related to its role in ribosome assembly or translation. Our structural data in fact provides concrete evidence that the isoform 1 of PA2G4 has a specific molecular role in pre-60S assembly.

### Maturation of the PTC orchestrated by NMD3 and GTPBP4

**Fig. 4:**
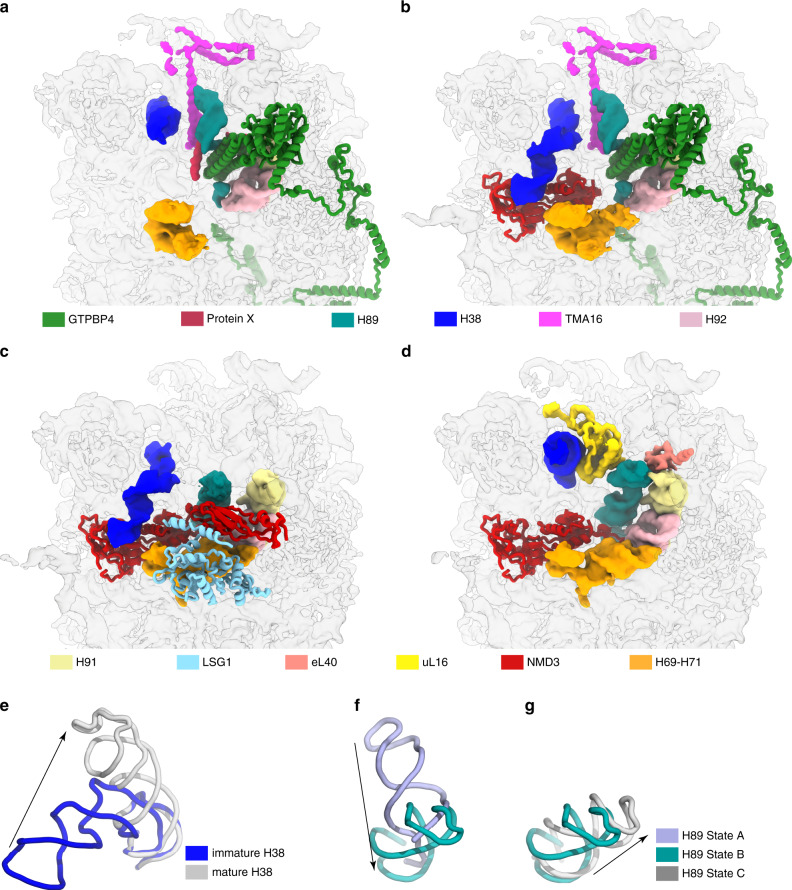
Progressive maturation of the PTC in NMD3-particles. **a-d** Conformational changes of the PTC components from state pre-A to state C. Changed components are highlighted in different colors (H38, H89, H92, H91, H69-H71, uL16 and eL40) and the four maps are shown in transparent surface representation. The mode of NMD3-NTD in state B (**c**) was derived from the structure of yeast NMD3 (PDB 6RZZ)^23^. **e** Conformational change of H38 from the immature (blue) to mature state (gray). **f**, **g** Continuous conformational changes of H89 during the maturation of the PTC.

From state pre-A to C, a clear pattern of step-wise conformational maturation of the PTC region could be seen, including PTC helices H89-93, as well as rRNA helices next to the PTC, such as H69-71 and H38 (Fig. 4a–d). Notably, these sequential changes on the 28S rRNA are coordinated with the release and binding of different sets of factors/ribosomal proteins.

In state pre-A and A, similar to the yeast pre-60S Nog2-particles^32^, the NTD of GTPBP4 is inserted into the two strands of H89, which places H89 in a completely different position compared to its mature form. In contrast to the yeast structures, this distinct conformation of H89 is accompanied by the binding of TMA16 (Fig. 4a,b), as well as an additional factor X in state pre-A (Fig. 1a, Supplementary Fig. 4a). NMD3-NTD is highly flexible in state A and points towards the 40S direction (Fig. 3a). In addition, the PTC-interacting β4-β5 loop from the RNA-binding domain I (eL22-like domain) of NMD3 is also flexible. The immature H92 interacts with one strand of the split H89, and the latter directly excludes the incoming β4-β5 loop from contacting the PTC (Fig. 3f). As the particles transit from state A to B, a near-mature state of H89 is reached (Supplementary Movie 3) upon the release of GTPBP4 and TMA16 (Fig. 4f). In this state, NMD3-NTD becomes ordered likely due to the interaction with LSG1 (Fig. 4c)^23,24^, and in particular, the specific interaction between the β4-β5 loop and the PTC (H92 and H90) is also established. This PTC-interacting motif of NMD3 is conserved across species: an invariant histidine residue (H232) directly interacts with the base of C4519 (Fig. 3g). Subsequent release of LSG1 in state C and integration of eL40, as well as the release of LLPH from cleft between H91 and H95 (Supplementary Fig. 10a,b), together lead to the final maturation of H89 in its final form (Fig. 4d,f,g, Supplementary Movie 3), in which a base stacking interaction between the tips of H89 and H91 is achieved.

Importantly, the maturation of H92 is coupled with the central helices H69-71. In all four states, H69 is in near mature conformations, but the tip of H69 has up to 8-Å structural difference among these states (Supplementary Fig. 11a–c). Density of H71 is not well resolved in state pre-A, and only a short helix of H71 could be seen in state A. In contrast, a well-ordered conformation of H71 is achieved in state B/C (Supplementary Fig. 11a–c). Also highly similar to the assembly of prokaryotic pre-50S particles^43,44^, the rigidification of H71 in state B and C is coupled with the stabilization of H92, through a evolutionarily conserved tripartite interaction among nucleotide residues from H92 (C4502 and U4498) and H71 (U3802) (Supplementary Fig. 11d,e).

H38, another functionally essential rRNA helix, is also associated with sequential maturation events. In state pre-A, H38 is roughly in its mature-like position, but the distal portion is highly flexible (Fig. 4a). Similar as observed in yeast pre-60S particles, H38 is deflected by 55° in states A and B, and this conformation is stabilized through extensive interactions with NMD3 (Fig. 4c,e, Supplementary Fig. 12a,b,d). The ~100 amino-acids at the C-terminus of NMD3 is poorly resolved in our structures, as well as in previous yeast pre-60S particles^23,24,33,34^. A new finding is that a stretch of evolutionarily conserved basic residues (R410 to K420) immediately downstream the RNA-binding OB domain of NMD3, not sufficiently resolved for atomic modeling though, is clearly seen to be embedded in the major groove of H38 in states A and B (Fig. 1a, Supplementary Fig. 12e,f). The major groove inserting motif of NMD3 (Supplementary Fig. 12g) likely plays an essential role in deforming H38. Notably, uL1 also contributes to this distortion of H38 as well: a basic residue R122 is seen to contact C1768 (Supplementary Fig. 12c). From state B to state C, H38 shifts from the distorted conformation to a near mature state (Supplementary Movie 4), which is coupled with the binding of uL16. It has to be noted that the P-site loop of uL16 in still flexible in state C. This is due to a competition between the β4-β5 loop of NMD3 and the P-site loop of uL16 in their accommodation into the PTC (Fig. 3h)^23^.

### Peptide exit tunnel maturation by GTPBP4 and ZNF622

**Fig. 5:**
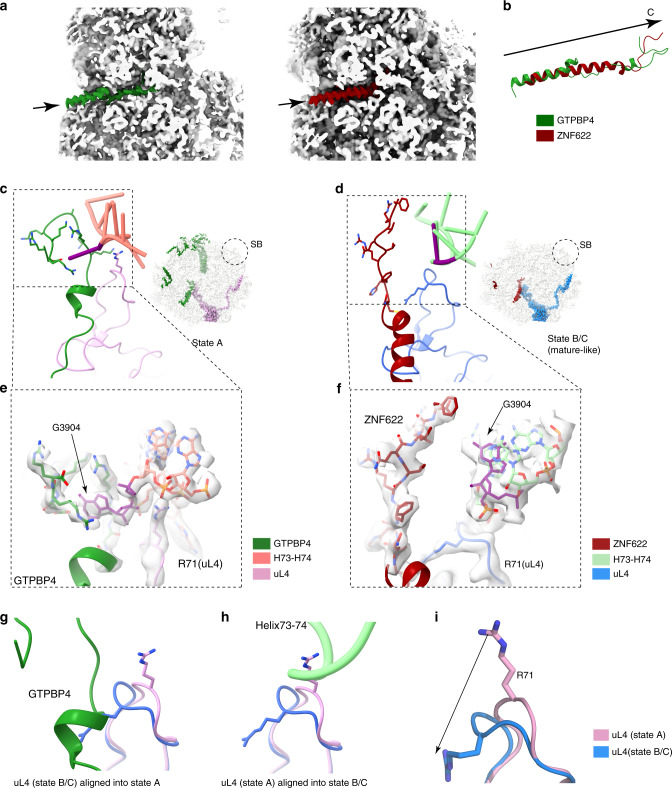
Insertion of the C-terminal tails of GTPBP4 and ZNF622 in the PET. **a** Cross-section views showing the insertion of the C-terminal tails of the two factors into the PET of the pre-60S structures (GTPBP4 in state A, ZNF622 in state B/C). The peptide tunnel exit is labeled by an arrow. **b** Superimposition of C-terminal tails of GTPBP4 and ZNF622 in the same view as in **a**. **c**, **d**, The C-terminal end of two factors interact with the PTC-PET components (H73-H74 linker and uL4) in state A (**c**) and state B/C (mature like, **d**) respectively. **e**, **f**, Zoom-in views on H73-H74 linker related to (**c**) and (**d**). The C-terminal ends of GTPBP4 and ZNF622 are shown in stick models. Conformation change of G3904 in two states are highlighted. **g**, **h**, Superimposition of uL4 in GTPBP4-bound and ZNF622-bound states. The mature position of R71 (blue, state B/C) clashes with GTPBP4 (**g**), whereas its immature conformation (pink, state A) is incompatible with H73-74 (**h**). **i** Structural comparison of the conserved loop in uL4 in the two states. R71 in the loop is shown.

Nascent peptide exit tunnel (PET) is an important functional center of the ribosome, which is composed of rRNA components from five of the six domains of the 28S rRNA. The construction of PET starts very early in nucleolar stages^45^, and yeast Nog1, Rei1 and Reh1 were found to continuously probe the tunnel through the insertion of their C-terminal sequences into the tunnel^32,33,37^. In our structures, we also found that two factors, GTPBP4 and ZNF622 play similar roles in the human pre-60S particles. The tunnel is occupied by the C-terminal extension (CTE) of GTPBP4 in pre-A and A, and by the CTE of ZNF622 in states B and C (Fig. 5a,b).

The pattern of interactions between these two factors and the PET components is highly similar across species. The tunnel portion of GTPBP4 is extremely conserved, and a number of invariant basic residues are seen to interact with the rRNA components (such as H35, H50, H93) in a conserved manner (Supplementary Fig. 13a–e). To name a few, R634 (the very C-terminal residue) directly contact the base of C1594 through polar interaction, while H620 stacks with A1600 of H35 (Supplementary Fig. 13a–e). In these two examples, the rRNA residues are in mature positions, whereas the stacking interaction between R626 and U4556 stabilizes the latter in a flipped position compared to its mature position in state B. In contrast, the tunnel portion of ZNF622 is less conserved than that of GTPBP4 (Supplementary Fig. 13f–j). However, in the presence of ZNF622, residues of the wall components are all in their mature forms. For example, the very C-terminal end of ZNF622 reaches the PTC and the terminal F477 clashes with superimposed U4556 (H73-H93 linker) in state A, leading to a conformational transition of U4556 from its immature (state A) to mature position (state B) (Supplementary Fig. 13f–j). Near the tunnel exit, both GTPBP4 and ZNF622 interact with H50 and H24, but through different physical contacts (Supplementary Fig. 14). Two residues of GTPBP4, Q597 and N601, interact with two consecutive bases of H50 (G2416 and A2417) in state A (Supplementary Fig. 14a), whereas only one contact is seen between Q449 of ZNF622 and A2417 in state B (Supplementary Fig. 14b). As to H24, the base of G412 has a similar interaction either with M592 of GTPBP4 or with M440 of ZNF622 in the two states, but G413 is only seen to interact with Y447 of ZNF622 in State B. It has to be noted that these assembly factor specific interactions of H50 and H24 appear to well correlate with its maturation status: G2416 of H50 in state A is in an immature position stabilized by Q597 of GTPBP4 (Supplementary Fig. 14c), whereas G413 of H24 in state B has acquired a mature conformation through stacking with Y447 of ZNF622 in state B (Supplementary Fig. 14d).

As the tunnel wall components, uL4 and uL22 participate in early nucleolar construction of the tunnel^46^, and some of their internal loops form constriction sites in the tunnel. In both the GTPBP4 and ZNF622 bound states, the constriction loops of uL4 (residues 65-89) (Fig. 5c,d) and uL22 (Supplementary Fig. 15a,b) are well ordered in mature-like conformations, but with subtle differences. The loop of uL4, as exemplified by the long sidechain of R71 (Fig. 5i), is seen to have a very dramatic change transiting from state A to state B/C (Fig. 5g–i), which is coupled with the release of the base of G3904 (linker of H73-74) from a deep pocket in GTPBP4 (Fig. 5e,f). R128 of uL22, also has a local sidechain reorientation from state A to state B/C (Supplementary Fig. 15c–e). Through these structural comparisons, a clear pattern is that subtle, local structural rearrangements on wall components continue to occur during transition from state A to C, although the general tunnel construction has already completed in the GTPBP4 bound state.

### Coordination between the PTC maturation and PET completion

**Fig. 6:**
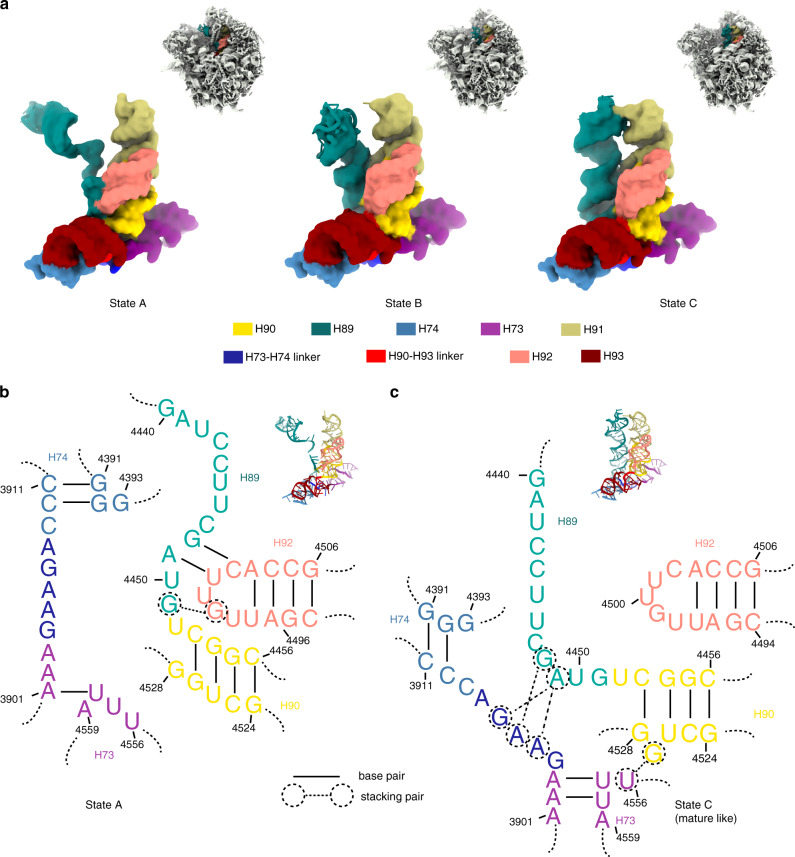
Coordination between the PTC maturation and tunnel construction. **a** Overviews of pre-60S density maps (state A, B and C) shown in surface representation, with components of the PTC-PET individually colored. Zoom-in views on the PTC-PET components (H89 to H93, H90-H93 linker and H73-H74 liker) are shown in lower panels. **b**, **c** Diagrams of secondary structures of the PTC-PET components in the immature state A (**b**) and mature-like state B/C (**c**), highlighting the contrasting base-pair and base-stacking interactions between the two states. Base-pairings are indicated by black lines and stacking interactions by dashed lines and circles.

GTPBP4 is a special factor that binds to both the PTC and PET of the pre-60S particles, making it a candidate for coordinating the conformational maturation of these two functional centers^31^. Indeed, comparison of state A with state C reveals a conformational switch of H89 that likely couples the maturation of the PTC with the final completion of the tunnel wall (Fig. 6a–c).

**Fig. 7:**
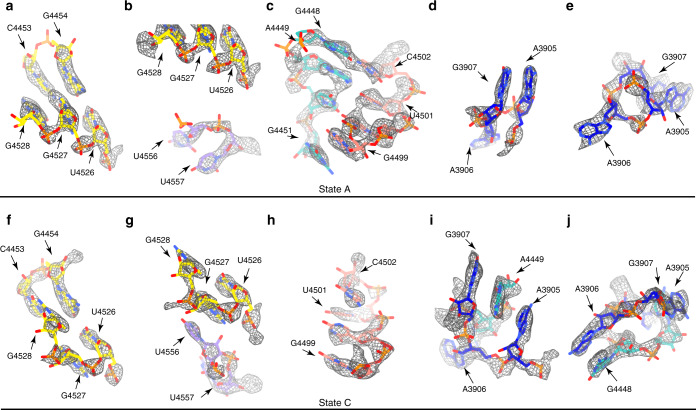
Detailed base interactions in the PET-PTC components upon transition from state A to state B/C. **a–e** Selected rRNA residues of the PTC-PET components in the immature state A is shown in stick models, superimposed with local densities. Residues from H90, H73-H93 linker, H89, H92, H73-H74 linker are colored yellow, purple, cyan, pink and blue, respectively. Non-native (immature conformation) base-pairings between C4453 (H90) and G4527 (H90), between G4448 (H89) and C4502 (H92), and between A4449 (H89) and U4501 (H92) are highlighted in **a** and **c**. **f**–**j** Same regions of mature -like state C as in **a**–**e** are shown. Base flip of G4527 (**f**) in state C (mature like) disrupts the base-pair in **a**. Native base stacking formed in state C between G4527 (H90) and U4556 (H73-H93 linker) is shown in **g**. Multiple base stacking interactions formed between residues of H89 and H73-H74 linker are highlighted in **i**, **j**.

In state A, the split strand of H89 is seen to form a pseudoknot with the tip of H92, through two canonical base pairs between G4448 and C4502, and between A4449 and U4501, and one base stacking between G4451 and G4499 (Figs. 6b and 7c). Notably, these interactions are not present in the mature 60S subunit, suggesting that they might be important for maintaining such an abnormal position of H89. In this state, the H73-74 linker of the wall component is also in an immature conformation (Fig. 7d,e): Particularly, the base of G3904 is embedded in a pocket formed by GTPBP4-CTE (Fig. 5e). It is worth mentioning that structural details of immature PTC configuration are considerably different from that of the yeast pre-60S particles, as the two base pairs between H89 and H92 were not seen in yeast pre-60S structures.

Upon transition to state B/C (after GTPBP4 release), the same strand of H89 which contacts H92 in state A, instead, interacts with H73-74 linker (G3904-A3908) through extensive stacking interactions: A4449 now inserts its base between those of G3907 and A3905, and the adjacent G4448 stacks with A3906 (Figs. 6c and 7i,j). These newly established interactions are accompanied by local structural rearrangement of the wall components on adjacent or surrounding residues from rRNA or r-protein, including the release of G3904 from GTPBP4, and repositioning of the L4 loop (Fig. 5f–h). Along with this conformational switch of H89, an interesting structural rearrangement also occurs on H90. C4453 switches its base-pair partner from G4527 (state A) (Figs. 6c and 7a) to adjacent G4528 (state C) (Figs. 6c and 7f), leading to the bulging of G4527 to form a base stacking between G4527 and U4556 of H73-H93 linker (Figs. 6c and 7g). In addition, a substantial rearrangement of H73 takes place as well. U4558, which basepairs with A3901 in state A, alters to basepair with A3902 in state C (Fig. 6c).

These observations suggest that structural remodeling of the PTC is coupled with the final completion of the tunnel wall. This coordination is through a conformational switch of H89, which is likely triggered by the release of GTPBP4. In this regard, as a P-loop GTPase, GTPBP4 is a perfect candidate which could work as a typical molecular switch by sensing a specific structural maturation event. Upon GTP hydrolysis, the release of GTPBP4 further initiates downstream assembly cascades.

In summary, we present a collection of human pre-60S structures sampled through a nuclear export adapter NMD3, representing structural snapshots of pre-60S particles immediately before and after passing through nuclear pore complex. The rich structural details in our structures serve as a valuable resource for dissecting the general mechanism, as well as the species-specific features of human ribosome assembly. In addition, it offers a more relevant structural framework to study the human diseases related to assembly dysfunction, particularly in late steps concerning the PTC maturation, such as Shwachman-Diamond Syndrome^47^.

## Methods

### Cell culture

HEK293FT cells (Invitrogen, R70007) were cultured in DMEM medium (Hyclone, SH30243.01B) supplemented with 10% fetal bovine serum (FBS) (Biological Industries, 04–001–1 A), 1x glutaMAX (Gibco, 35050-061), 100 U/ml penicillin and 10 mg/ml streptomycin (Solarbio, P1400), at 37 °C in a 5.5% CO_2_ humidified atmosphere for stable cell line establishment. When establishment completed, cells were transferred to flanking bottles in SMM 293-TI (Sino Biological inc, M293TI-1), containing 1% FBS, 1x glutaMAX, 100 U/ml penicillin and 10 mg/ml streptomycin for purification. Cell lines were not authenticated or tested for mycoplasma contamination.

### Stable cell line establishment

To generate stable cell line expressing NMD3 with an affinity tag at the C-terminus, the DNA sequence encoding NMD3-TEV-3FLAG was amplified from Human NMD3 cDNA library using PCR. The target sequence was cloned into a lentiviral expression vector pLVX-AcGFP1-N1(ClonTech) using ClonExpress II One Step Cloning Kit (Vazyme). Cell transfection was adapted from a previous work with slight modifications^48^. The cells were allowed to grow to 80% confluency and transfected with NMD3-tev-3XFLAG in pLVX-AcGFP1-N1, pMD2.G and psPAX2 (from Dr. Li Yu, Tsinghua University) in mass ratio 2:2:3 using Polyethylenimine (PEI) (Linear MW 25,000, Polysciences.Inc, 23966-1), which was added to the plasmid-DMEM solution drop by drop to reach a PEI to total plasmid mass ratio of 3:1 and mixed thoroughly. 48 h after transfection, medium, containing target lentivirus, was collected to infect new HEK293FT for 48 h with 8 μg/ml polybrene. Selection was performed with 2 μg/ml puromycin (Ameresco, J593-25MG). When selection finished, the adherent cell line was tamed to suspension via reducing concentration of FBS from 10% to 1% gradually in SMM 293-TI, containing 1x glutaMAX, 100 U/ml penicillin and 10 mg/ml streptomycin.

### Purification of pre-60S NMD3 particles

Pelleted (1,850 g) for 10 min at 4 °C and washed once with PBS, cells were re-suspended in lysis buffer [50 mM HEPES (pH = 7.8), 60 mM KCl, 5 mM MgOAc, 0.05% NP-40, 1x cocktail Protease Inhibotor, 1 mM NaF, 5% glycerol and 40U RNasIN Ribonuclease Inhibitor (N2111, Promega)] and lysed by high pressure cell disruptor (JNBIO). The lysate was cleared for 30 min at 25,000 g in JA25.50 rotor (Beckman Coulter). Supernatants were collected and incubated with equilibrated ANTI-FLAG M2 Affinity Agarose Gel (Sigma-Aldrich) for 2 h, followed by five times washing with 20 c.v. (column volume) wash buffer [50 mM HEPES (pH = 7.8), 60 mM KCl, 5 mM MgOAc, 0.05% NP-40, 1x cocktail Protease inhibitor, 1 mM NaF] (100 c.v. totally) and eluted with 2 mg/ml 3XFLAG peptide in 5 c.v. wash buffer. Eluate was loaded onto a sucrose cushion buffer [40 mM HEPES (pH = 7.8), 90 mM KCl, 5 mM MgOAc and 30% w/v sucrose] in volume ratio of 10:9, and pre-60S particles were concentrated via centrifugation at 100,000 rpm in a TLA100 rotor (Beckman Coulter) for 23 mins and re-suspended in ribosome buffer [40 mM HEPES (pH = 7.8), 90 mM KCl, 5 mM MgOAc].

### Cross-Linking Mass Spectrometry (CXMS)

About 10 μg of purified ribosome complexes in a volume of 10 μl was cross-linked by both DSS and BS^3^ both at a final concentration of 0.5 mM, incubated for 30 min at room temperature. Then, a final concentration of 0.2 mM KArGO was added to the samples. The cross-linked proteins were precipitated with ice-cold acetone at −20 °C overnight, resuspended in 8 M urea, 100 mM Tris, pH 8.5. After trypsin digestion at 37°C overnight, the peptides were desalted by ZipTip pipette tips (Merck Millipore). The LC-MS/MS analysis was performed on an Easy-nLC 1000 UHPLC (Thermo Fisher Scientific) coupled to a Q Exactive HF Orbitrap mass spectrometer (Thermo Fisher Scientific). Peptides were loaded on a pre-column (75 μm inner diameter, 4 cm long, packed with ODS-AQ 12 nm–10 mm beads from YMC Co., Ltd.) and separated on an analytical column (75 μm inner diameter, 13 cm long, packed with ReproSil-Pur C18-AQ 1.9 μm 120 A˚ resin from Dr. Maisch GmbH) using an linear gradient made with buffer A (0.1% formic acid) and buffer B (100% acetonitrile and 0.1% formic acid) as follows: 0–3 min, 0–5% B; 3–93 min, 5–30% B; 93–98 min, 30–95% B; 98–103 min, 95% B; 103–105 min, 95-0% B; 105–110 min, 0% B. The flow rate was 250 nl/min. The top 15 most intense precursor ions from each full scan (resolution 60,000) were isolated for HCD MS2 (resolution 15,000; NCE 27) with a dynamic exclusion time of 30 s. Precursors with 1+, 2+, 8+, above 8+ or unassigned charge states were excluded. In order to identify proteins in the sample, an additional LC-MS/MS analysis was performed without rejecting precursors of 2+ charge state, and the MS data were then searched against a human proteome database using pFind3^49^. A database containing the sequences of identified proteins was constructed for searching cross-linked peptides. pLink2^50^ was used to identify cross-linked peptides pairs with FDR < 5% at the spectrum level, E-value < 1E-7, # of spectrum >2.

### Cryo-EM specimen preparation

The pre-60S sample was diluted to a concentration of ~120 nM. Prior to sample freezing, holey carbon grids (Quantifoil R1.2/1.3) were coated with a thin layer of freshly prepared carbon and glow-discharged for 35 s in low level with plasma cleaner (PDC-32G-2, Harrick Plasma). 4-μl aliquots of diluted sample were loaded on grids, blotted for 1 s and plunge-frozen into liquid ethane via an FEI Vitrobot Mark IV at 8 °C and 100% humidity.

### Data collection and image process

Cryo-grids were screened in an FEI Talo Arctica and transferred to an FEI Titan Krios microscope operated at 300 kV for data collection. Images was acquired using SerialEM^51^ on a K2 Summit direct electron detector (Gatan) at a magnification of 130,000 (pixel size of 1.058 Å at the object scale), and with the defocus varying from −1.2 to −1.8 μm. Each micrograph was dose-fractionated to 32 frames with a dose rate of ~8 electrons per Å^2^ per second for a total exposure time of 8 s.

Four datasets were collected and processed following the same procedures. Movie stacks were summed for drift correction and dose weighting via MotionCorr2^52^. The CTF parameters were estimated with Gctf program^53^, and micrograph screening were performed manually with SPIDER^54^. Further image processing, including 2D and 3D classification, 3D auto-refine, CTF refinement and post-processing, was done with RELION 3.0 and 3.1^55^.

After 2D and 3D classification of the four batches of particles, all 60S classes were combined for further processing (with a binning factor 2) (Supplementary Fig. 1d). The resulting 110,000 particles were subjected to one round of 3D classification with four initial references (mature 60 S, 80 S, and two pre-60S maps from previous round of 3D classification). Only 4.4% particles were grouped in the mature-like class (no NMD3 and eIF6 density). Two major groups, class 1 (strong density of GTPBP4) and class 2 (in the absence of GTPBP4) account for 39.3% and 56.3% of all particles, respectively.

Class 1 shows mixed states of L1 stalk (open and close), indicating the presence of structural heterogeneity. 3D classification with reference mask focusing on GTPBP4 (‘NO’ in ‘Perform image align’ option) was first applied to remove those with weak or without GTPBP4 density. 34,700 particles were kept for high-resolution refinement (without binning). The final refinement with dose-weighted particles (with ‘CTF refinement’ in RELION 3.0) resulted in a 3.3-Å map (gold-standard FSC 0.143 criteria). Next, we applied particle subtraction focusing on a region including TMA16, L1 and surrounding components (removed the densities of other regions from the raw particles) to perform further 3D classification (into two classes). Two states were obtained, state pre-A (10,277 particles) and state A (21,489 particles), at resolutions of 3.6 Å and 3.5 Å, respectively (gold-standard FSC 0.143 criteria). After applying CTF refinement with default settings in RELION 3.1, the maps were further improved to 3.2 Å and 3.1 Å, respectively.

Class 2 displays mixed states of H38. Focused 3D classification (five classes) on regions of LSG1 and immature H38 was performed. Two populated states were obtained, with H38 in distinct positions. These two states, state B (18,819 particles) and state C (21,707 particles) were further refined (RELION 3.0) to 3.5 Å and 3.4 Å, respectively. After applying CTF refinement with default settings in RELION 3.1, the maps were further improved to 3.1 Å and 3.0 Å, respectively.

Density of PA2G4 and ES27 is relatively weak and fragmented in the map of class 2. To improve the local density of PA2G4, focused 3D classification (5 classes) was performed with a reference mask including regions of PA2G4 and ES27 using particles of class 2. One class (17,896 particles) with strong ES27 and PA2G4 density were used for the final refinement (3.8-Å). Further CTF refinement with RELION 3.1 improved the map (State D) to 3.3-Å resolution (gold-standard FSC 0.143 criteria). Local resolution maps were generated using ResMap^56^.

### Atomic model building and refinement

To facilitate modeling, the starting 3D models of NMD3, TMA16, GTPBP4, eIF6, GNL2 and RLP24 were generated using SWISS-MODEL^57^, and models of ZNF593 and ZNF622 were built using I-TASSER^58^. Multiple structures of yeast pre-60S particles were used as templates by these two programs (PDB 3JCT, 5H4P, 5APO, 5T62, 6N8L, 6QIK, 6RZZ, 6ELZ)^23,24,32–34,36,37^. PSIPRED^59^ was used to predict secondary structures for all these factors. These generated 3D models were docked into the density map by rigid-body fitting, followed by extensive manual rebuilding in COOT^60^. NMD3, TMA16, RLP24, ZNF593, eIF6 and GTPBP4 are well resolved, which enabled atomic modeling for the majority (or nearly entire) of their sequences. The visible portions of GNL2, tunnel portion of ZNF622, and LLPH are also well resolved and modeled. For modeling of LLPH, a poly-alanine model was first built in COOT, and several candidate human proteins (from BLAST analysis of yeast YBL028C against human protein database) were tested based on predicated secondary structures and side-chain density matching. For modeling of TMA16 (identified through CXMS), a homology model was first built based on the solution NMR structure of yeast TMA16 (YOR252W, PDB 2KKM) using SWISS-MODEL, and the final model of LLPH was by manual rebuilding in COOT. For protein X, unfortunately, CXMS experiments failed to reveal its identity. The α-helix of protein X (28 amino acids) is sufficiently resolved to determine its polarity. Therefore, extensive trial-and-error modeling of potential sequences from the mass spectrometry data (as well as the sequence of NMD3-NTD) were performed. In addition, a map-derived degenerate amino-acid sequence based method (CryoID)^61^ was also tried. None of these efforts worked and the identity of protein X remains unknown. PA2G4 in state D is not sufficiently resolved for atomic modeling. Instead, a crystal structure of human PA2G4 isoform1 (PDB 2Q8K)^26^ was fitted (rigid-body) in the map. For the same reason, homology models of LSG1, NMD3-NTD, ZNF622-NTD and MRTO4 (from SWISS-MODEL or I-TASSER) were also fitted in respective maps by rigid-body fitting to facilitate structural analysis and figure preparation.

For modeling the ribosomal components, relevant chains from models of the 80 S ribosome (PDB 6EK0 and 5AJ0)^62,63^ were fitted into the map in UCSF Chimera^64^ and modeled in COOT. For r-proteins, individual models were manually fitted in the map and adjusted in COOT. Specifically, the models of ribosomal RNAs (28 S, 5 S and 5.8 S) were based on 6EK0. In state A, after rigid-body fitting, fragments of 1740-1790 (H38), 3964-4067 (L1 stalk), 3753-3820 (H69-71) were cut off. The model of the immature H38 from the yeast structure (PDB 6N8K)^24^ was fitted into the map of state A, adjusted and mutated to human nucleotide sequence using COOT. For H89, residues 4392-4396 were manually rebuilt with COOT, and residues 4403-4435 (H89) were similarly built using the yeast fragment as the initial model. For residues of 4448-4536 (containing H90-H93), 1855-1878 (H39), 3898-3910 (H73-H74), 4585-4626 (H95), corresponding models were fitted manually via rigid-body fitting in COOT. In State B, models of rRNAs were similarly built and the tip of H38, in contrast to that in state A, is in sufficient resolution for atomic modeling. In State C, ribosomal components closely resemble their mature conformations. uL16 and eL40 were modeled in state C, with the P-loop of uL16 removed from the model.

In each of these modeling tasks, extensive rounds of model refinement using real-space refinement in PHENIX^65^ (with secondary structure, geometry and base pair constraints applied) and manual rebuilding in COOT were applied. The atomic models were cross-validated according to a published method^66,67^. In short, the coordinates of the four models were randomly displaced by 0.1 Å via the PDB tools in PHENIX. The modified models were refined against the Half1 maps of the respective datasets. The refined model from the Half1 map was compared with the maps of Half1, Half2 and the final map in Fourier space to produce three FSC curves: FSC_work_ (Model vs Half1 map), FSC_free_ (Model vs Half2 map) and FSC_merge_ (Model vs Final map) (Supplementary Fig. 2a–d). As indicated by these curves, the agreement between FSC_work_ and FSC_free_ indicated that four model were not overfitted. The final models were evaluated using Molprobity^68^, and statistics of the model evaluation was provided in Supplementary Table 1.

### Statistics and reproducibility

Purification and sample preparation of native NMD3 pre-60S particles were repeated four time with similar results. Cryo-EM data was collected from two different grids with comparable results. No statistical analysis, other than those embedded in image processing, has been applied throughout the work.

### Reporting summary

Further information on research design is available in the Nature Research Reporting Summary linked to this article.

## Supplementary information

Supplementary Information

Peer Review File

Reporting Summary

Description of Additional Supplementary Files

Supplementary Movie1

Supplementary Movie2

Supplementary Movie3

Supplementary Movie4

## Data Availability

The data that support this study are available from the corresponding author upon reasonable request. The cryo-EM maps and atomic coordinates of the state pre-A, A, B, and C have been deposited in the EMDB and PDB databases with accession codes EMD-0964 [https://www.ebi.ac.uk/pdbe/entry/emdb/EMD-0964], EMD-0978 [https://www.ebi.ac.uk/pdbe/entry/emdb/EMD-0978], EMD-0963 [https://www.ebi.ac.uk/pdbe/entry/emdb/EMD-0963], EMD-0948 [https://www.ebi.ac.uk/pdbe/entry/emdb/EMD-0948] and 6LSS [10.2210/pdb6LSS/pdb], 6LU8 [10.2210/pdb6LU8/pdb], 6LSR [10.2210/pdb6LSR/pdb], 6LQM [10.2210/pdb6LQM/pdb], respectively. The CXMS data have been deposited to the ProteomeXchange Consortium (http://proteomecentral.proteomexchange.org) via the iProX partner repository^69^ with the dataset identifier IPX0002087000 [https://www.iprox.org/page/project.html?id=IPX0002087000] and PXD019025 [http://proteomecentral.proteomexchange.org/cgi/GetDataset?ID=PXD019025]
