# Supplementary material for: Structural snapshots of human pre-60S ribosomal particles before and after nuclear export
Source: Nat Commun. 2020 Jul 15;11:3542. doi: 10.1038/s41467-020-17237-x (PMC7363849; doi:10.1038/s41467-020-17237-x)
Supplement: Supplementary file 1 — Supplementary Information [file 41467_2020_17237_MOESM1_ESM.pdf]

Supplementary information

Structural snapshots of human pre-60S ribosomal particles before and after nuclear export

Xiaomeng Liang[1,2], Mei-Qing Zuo[3,4,5], Yunyang Zhang[2], Ningning Li[2], Chengying Ma[2], Meng-Qiu Dong[4,5], Ning Gao[2, *]

[1] State Key Laboratory of Membrane Biology, School of Life Science, Tsinghua University, Beijing 100084, China.

[2] State Key Laboratory of Membrane Biology, Peking-Tsinghua Joint Centre for Life Sciences, School of Life Sciences, Peking University, Beijing 100871, China.

[3] College of Biological Sciences, China Agricultural University, Beijing 100193, China.

[4] National Institute of Biological Sciences, Beijing 102206, China.

[5] Tsinghua Institute of Multidisciplinary Biomedical Research, Tsinghua University, Beijing 100084, China.

*Correspondence to: gaon@pku.edu.cn

This PDF includes

Supplementary Figures 1-15
Supplementary Tables 1-2

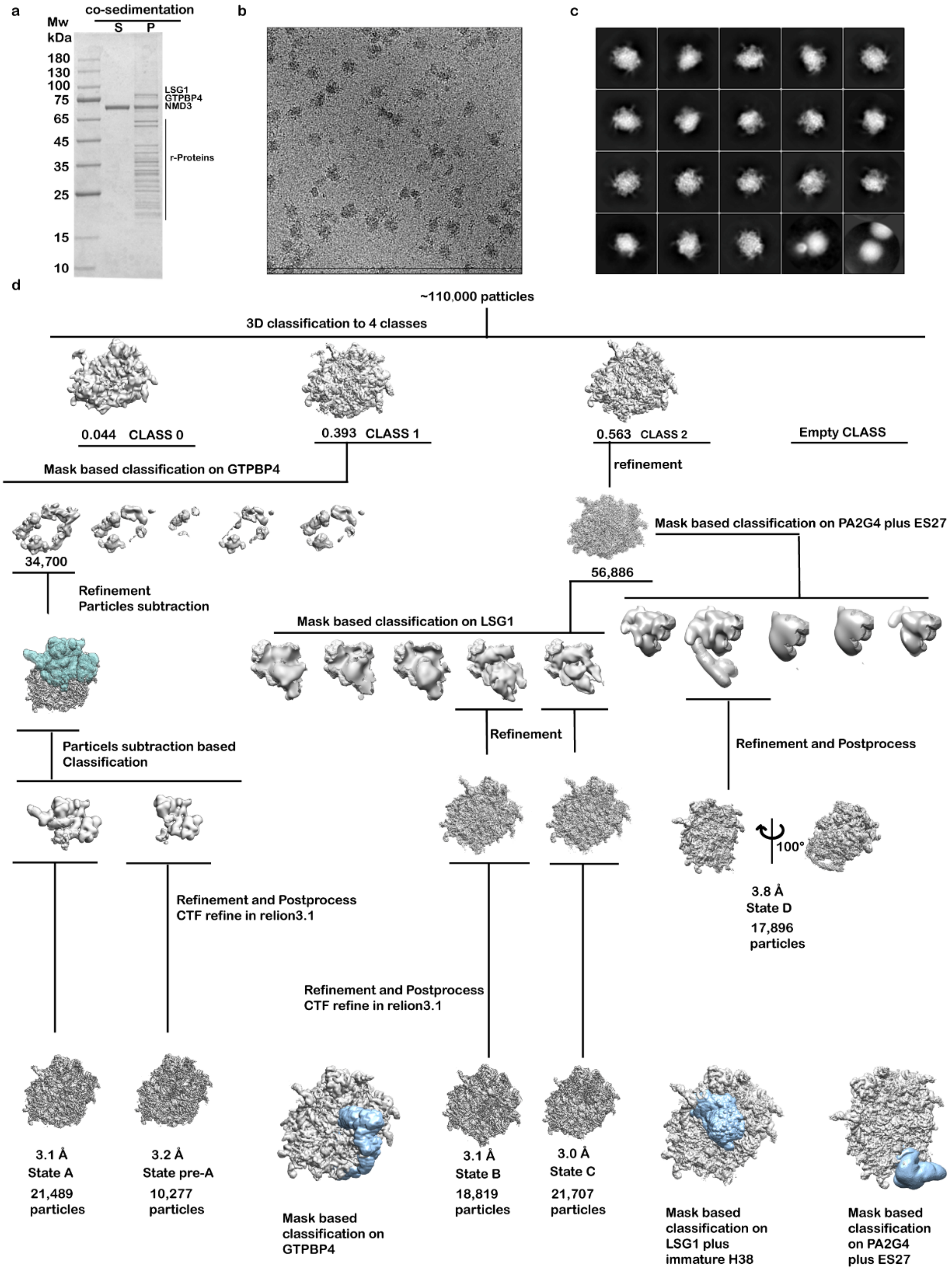
a
co-sedimentation
Mw kDa
S
P
180
130
100
75
65
45
35
25
15
10
LSG1
GTPBP4
NMD3
r-Proteins
b
c
d
~110,000 patticles
3D classification to 4 classes
0.044 CLASS 0
0.393 CLASS 1
0.563 CLASS 2
Empty CLASS
Mask based classification on GTPBP4
refinement
34,700
Refinement
Particles subtraction
Mask based classification on PA2G4 plus ES27
56,886
Mask based classification on LSG1
Particels subtraction based Classification
Refinement
Refinement and Postprocess
100°
3.8 Å
State D
17,896 particles
Refinement and Postprocess
CTF refine in relion3.1
Refinement and Postprocess
CTF refine in relion3.1
3.1 Å
State A
21,489 particles
3.2 Å
State pre-A
10,277 particles
Mask based classification on GTPBP4
3.1 Å
State B
18,819 particles
3.0 Å
State C
21,707 particles
Mask based classification on LSG1 plus immature H38
Mask based classification on PA2G4 plus ES27

Supplementary Fig. 1 | Sample purification and cryo-EM data process of human NMD3-particles.

a, Affinity purification and concentration by co-sedimentation of human pre-60S particles isolated via FLAG tagged NMD3. Supernatant (S) was discarded and Pellet (P) was used for cryo-EM analysis.

b, A representative micrograph of NMD3- particles in vitrified ice.

c, Representative 2D class averages of cryo-EM particles.

d, All datasets were processed following a general 2D and 3D classification scheme (See methods for details). Approximate 110,000 pre-60S particles (after 2D classification) were pooled for processing. 3D classification (into four groups) yielded two major pre-60S classes (class 1 and class 2) and one mature-like class (class 0). Focused classification (masked based) was perform on regions of GTPBP4, LSG1 or PA2G4+ES27 to improve the structural homogeneity. For separation of state A and pre-A, an additional round of density subtraction-based classification was used, in which only the regions of TMA16, L1 and surrounding components were kept in modified particles. For clarification, masks used in mask-based classification are also shown in the lower panels.

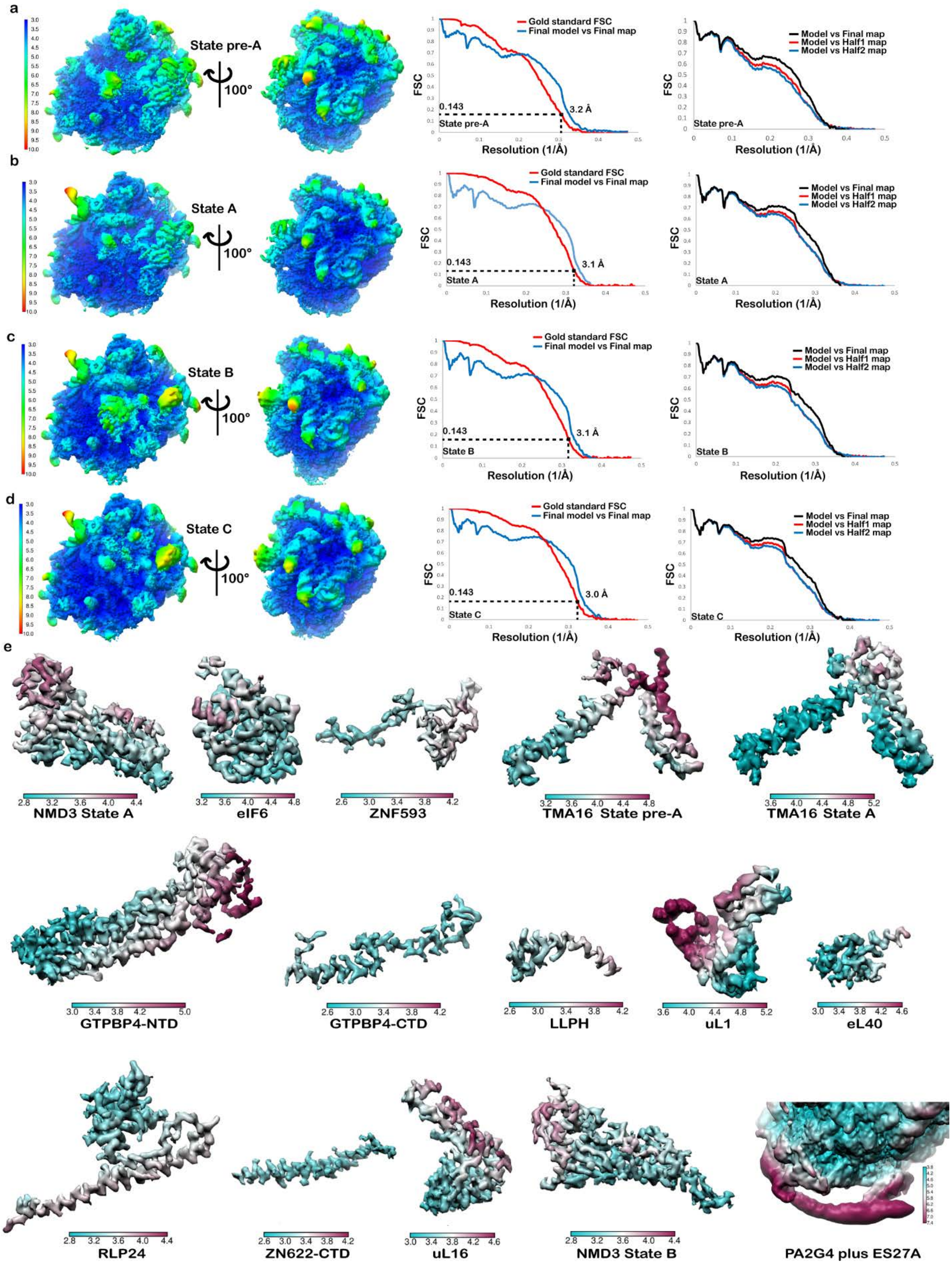
a
State pre-A
100°
Gold standard FSC
Final model vs Final map
0.143
3.2 Å
State pre-A
FSC
Resolution (1/Å)
Model vs Final map
Model vs Half1 map
Model vs Half2 map
b
State A
3.1 Å
c
State B
3.1 Å
d
State C
3.0 Å
e
NMD3 State A
eIF6
ZNF593
TMA16 State pre-A
TMA16 State A
GTPBP4-NTD
GTPBP4-CTD
LLPH
uL1
eL40
RLP24
ZN622-CTD
uL16
NMD3 State B
PA2G4 plus ES27A

Supplementary Fig. 2 | Resolution estimation and model validation of four major states.
a-d, cryo-EM maps of the four states are colored according to local resolution estimation (left two columns). Fourier shell correlation (FSC) curves for the final 3D density map after RELION-based post-processing (red, gold-standard FSC), and for the cross-examination between final atomic model and the 3D density map (blue, final refined model versus map) are shown (Middle column). FSC curves for the atomic model cross-validation (right column) (See Methods for details).
e, Local resolution maps of assembly factors and selected ribosomal proteins.

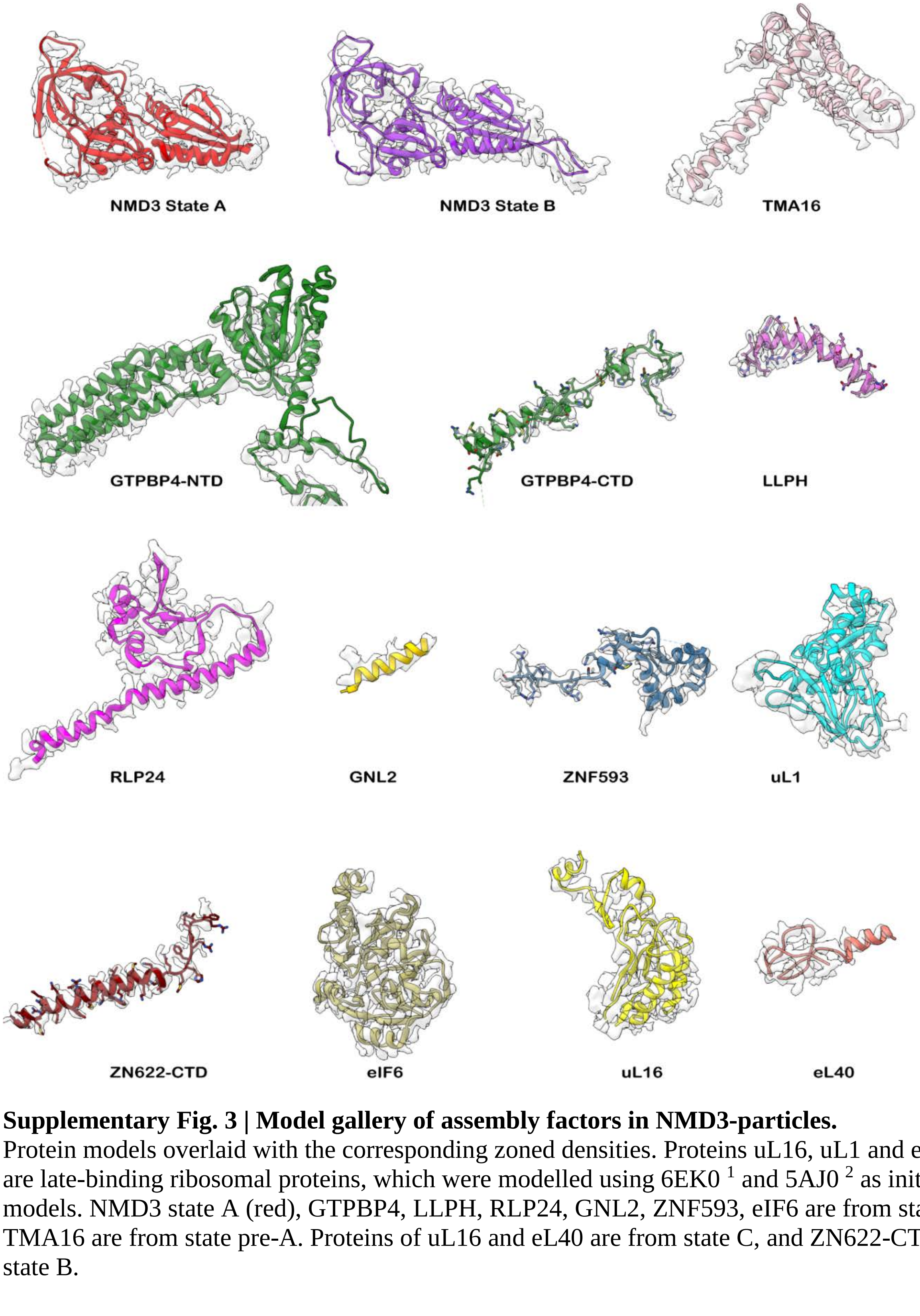

Supplementary Fig. 3 | Model gallery of assembly factors in NMD3-particles.
Protein models overlaid with the corresponding zoned densities. Proteins uL16, uL1 and eL40 are late-binding ribosomal proteins, which were modelled using 6EK0 [1] and 5AJ0 [2] as initial models. NMD3 state A (red), GTPBP4, LLPH, RLP24, GNL2, ZNF593, eIF6 are from state A. TMA16 are from state pre-A. Proteins of uL16 and eL40 are from state C, and ZN622-CTD from state B.

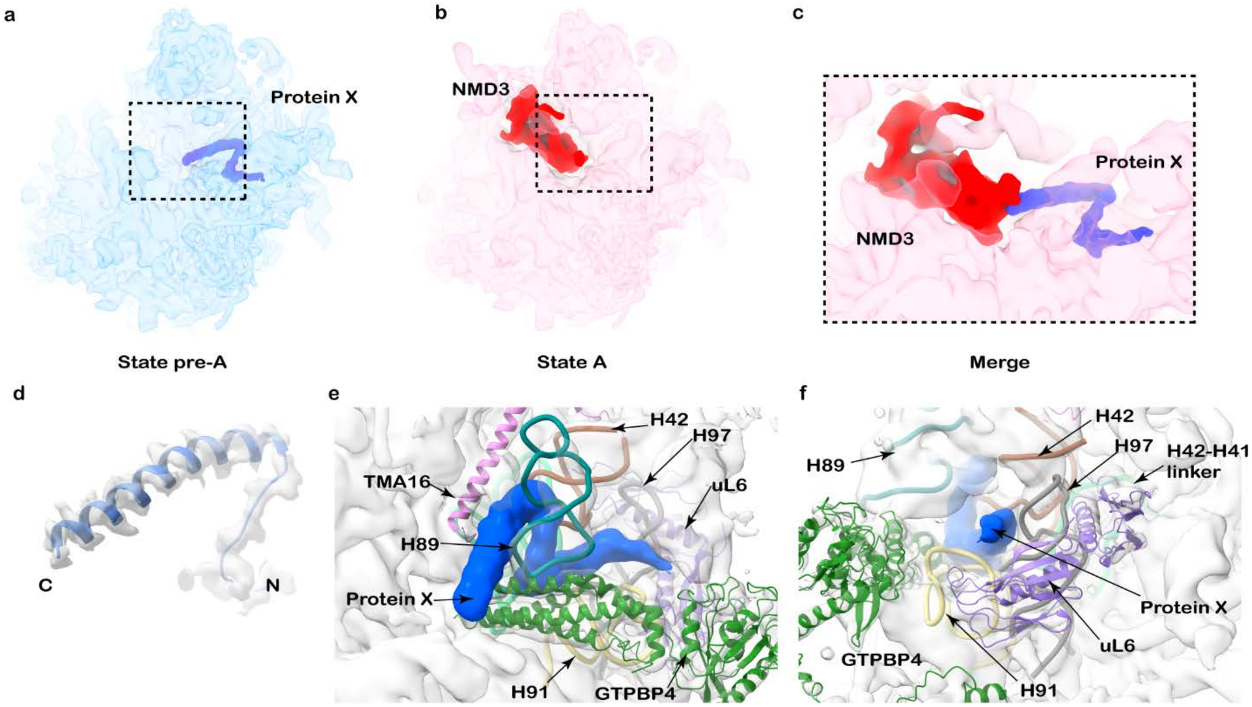

Supplementary Fig. 4 | ProteinX found in human pre-60S maps.

a-c, Superimposition of the maps of states pre-A (a) and A (b), with density of protein X and NMD3 in blue and red, respectively. The C-terminal end of protein X has a steric clash with the turning point of the NTD of NMD3 (c).

d, A poly-alanine model for the well resolved C-terminus of protein X (40 amino acids), including an α-helix (28 amino acids). The sequence identity of protein X was not determined.

e-f, Potential interactions between protein X and ribosomal components. The main chain of protein X could be traced up to 50 amino acids, from the well resolved C-terminal helix to a long N-terminal extension (NTE). This NTE extends underneath H89 and passes between H42 and H91(e). The very N-terminus ends in a surface exposed cavity surrounded by H42, H91, H97, H89 and uL6 (f).

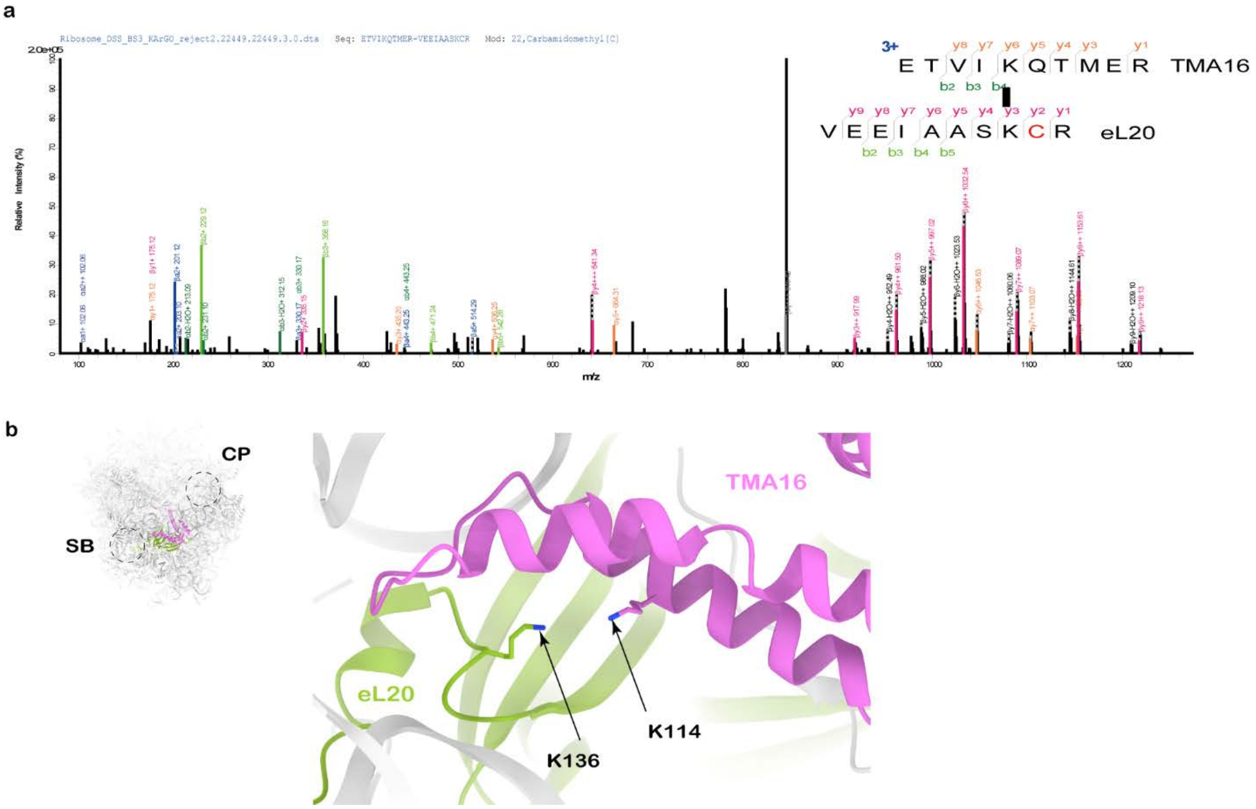

Supplementary Fig. 5 | New non-ribosomal proteins found in human pre-60S maps.
a, Representative HCD spectra showing the identification of crosslinking between TMA16 and eL20. The fifth residue (lysine) of the peptide ETVIKQTMER (TMA16) is crosslinked with the ninth residue (lysine) of the peptide VEEIAASKCR (eL20). C denotes carbamidomethylated cysteine. The precursor charge and m/z are shown in the spectra.
b, The two lysine residues of TMA16 and eL20 are displayed in the atomic model of state A.

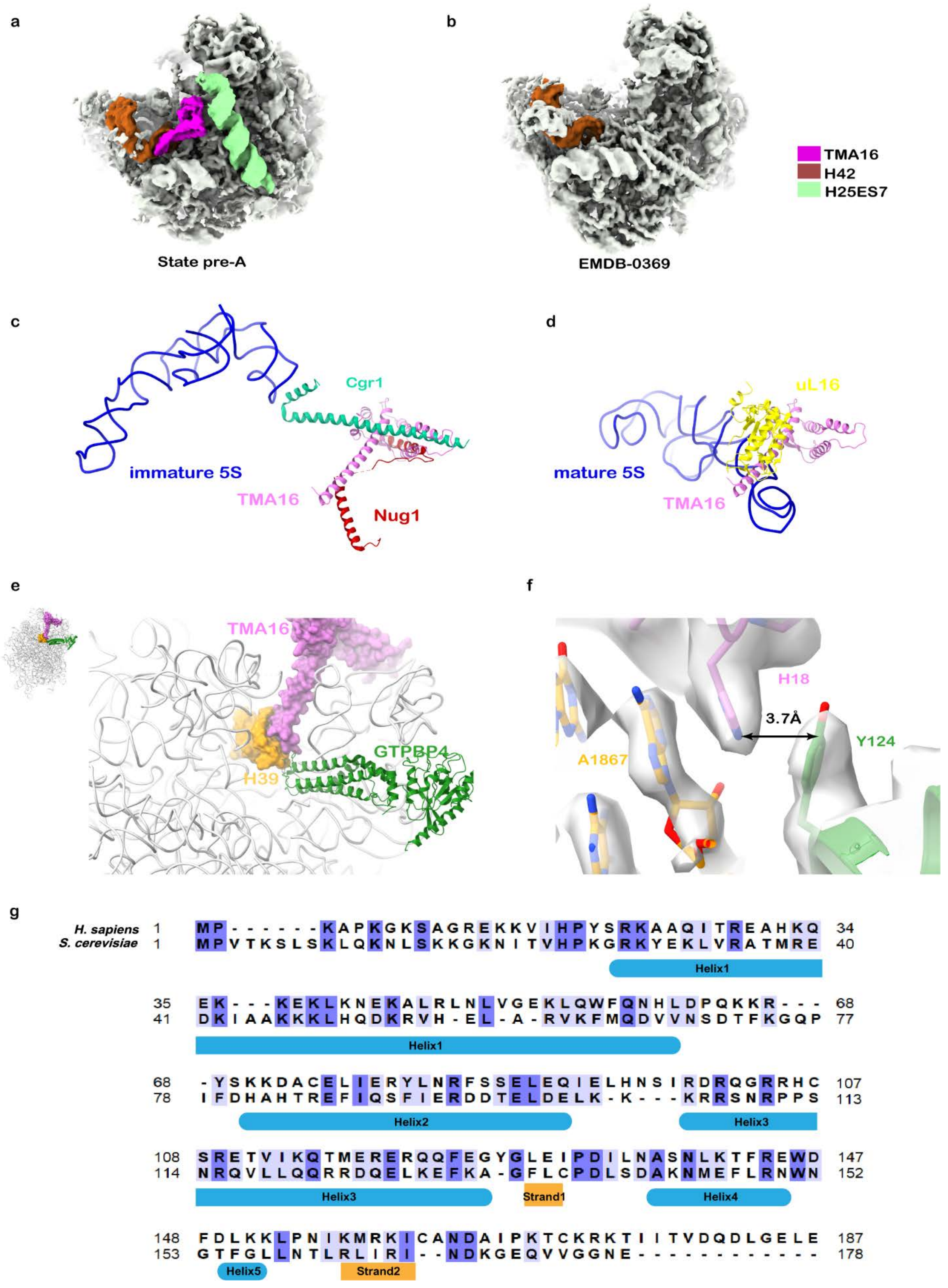
a
b
State pre-A
EMDB-0369
TMA16
H42
H25ES7
c
d
Cgr1
immature 5S
TMA16
Nug1
uL16
mature 5S
TMA16
e
f
TMA16
H39
GTPBP4
H18
3.7Å
A1867
Y124
g
H. sapiens 1 MP------KAPKGKSAGREKKVIHPYSRKAAQITREAHKQ 34
S. cerevisiae 1 MPVTKSLSKLQKNLSKKGKNITVHPKGRKYEKLVRATMRE 40
Helix1
35 EK---KEKLKNEKALRLNLVGEKLQWFQNHLDPQKKR--- 68
41 DKIAAKKKLHQDKRVH-EL-A-RVKFMQDVVNSDTFKGQP 77
Helix1
68 -YSKKDACELIERYLNRFSSELEQIELHNSIRDRQGRRHC 107
78 IFDHAHTREFIQSFIERDDTELDELK-K---KRRSNRPPS 113
Helix2
Helix3
108 SRETVIKQTMERERQQFEGYGLEIPDILNASNLKTFREWD 147
114 NRQVLLQQRRDQELKEFKA-GFLCPDLSDAKNMEFLRNWN 152
Helix3
Strand1
Helix4
148 FDLKKLPNIKMRKICANDAIPKTCKRKTIITVDQDLGELE 187
153 GTFGLLNTLRLIRI--NDKGEQVVGGNE------------ 178
Helix5
Strand2

Supplementary Fig. 6 | Structural analysis of TMA16 in the human pre-60S structure.
a-b, Comparison between the pre-60S structures from human (state pre-A) and yeast (EMD-0369) [3]. H42, TMA16 and part of H25ES7 are color-coded. As shown, the yeast map is empty in the position of TMA16.
c-d, TMA16 has steric clash with Nug1 **(c)**, Cgr1 **(d)** in the yeast pre-60S Nog2-particle (PDB 3JCT) [4] with immature 5S and uL16 **(e)** in state C with rotated 5S.
e, GTPBP4-NTD stabilizes the interaction between TMA16 and H39 of the 28S rRNA.
f, Y124 of GTPBP4-NTD further stacks with H18 of TMA16.
g, Sequence alignment of TMA16 between human and yeast.

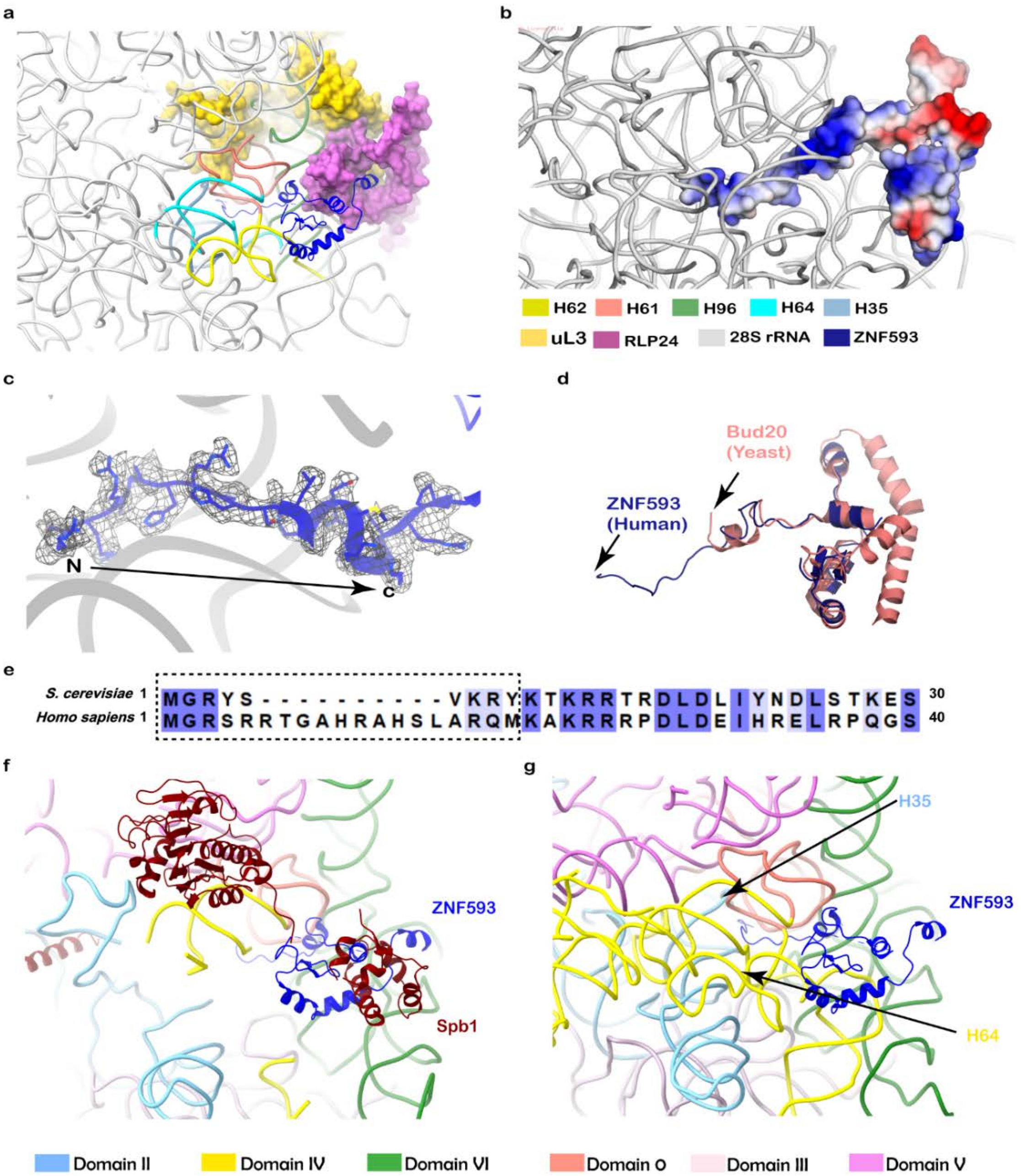

Supplementary Fig. 7 | Species-specific features of ZNF593.

a, Overview of ZNF593 in the structure of state A.

b, Positively charged N-terminal tail of ZNF593 is deeply inserted in a 28S rRNA cavity.

c, N-terminal tail of ZNF593 shown in stick model, superimposed with the segmented density map (mesh representation).

d, Superimposition between ZNF593 (blue, human) and Bud20 (salmon, yeast) (PDB 3JCT) [4]. The very N-terminus of the two factors are labelled with black arrows.

e, Sequence alignment of ZNF593 and Bud20 in the N-terminal regions.

f-g, Comparison of the pre-60S structures from the yeast State E and human State A. Structure of the yeast State E (PDB 6ELZ) [5] with superimposed ZNF593 (f). The same view of the structure of human State A (f). H64 and H35 in the human 28S rRNA are labelled. rRNA domains are highlighted in different colors.

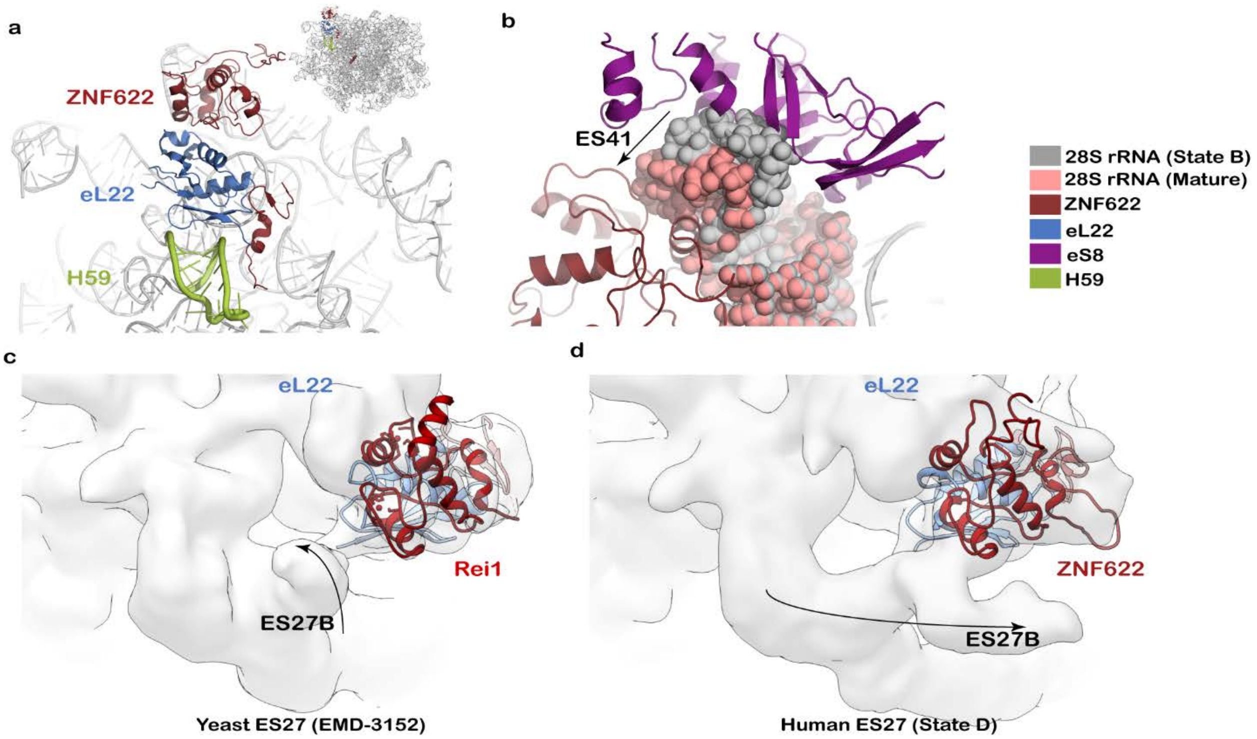

Supplementary Fig. 8 | Structures of ZNF622-NTD and in the human pre-60S particles.
a, ZNF622-NTD interacts with eL22 and H59.
b, Comparison of the ES41 conformations in state B and in the 80S ribosome (6EK0) [1]. The model of eS8 from the 80S ribosome (from 6EK0) is highlight in purple.
c, Densities of ES27 and Rei1 in the yeast pre-60S structure (EMD-3152) [6] . In the yeast map, the shorter branch of ES27B points to the 40S direction.
d, Densities of ES27 and ZNF622-NTD in the human pre-60S State D. The longer branch of ES27B in the human structure points to the PET (similar as ES27A) and display a specific contact with ZN622-NTD.

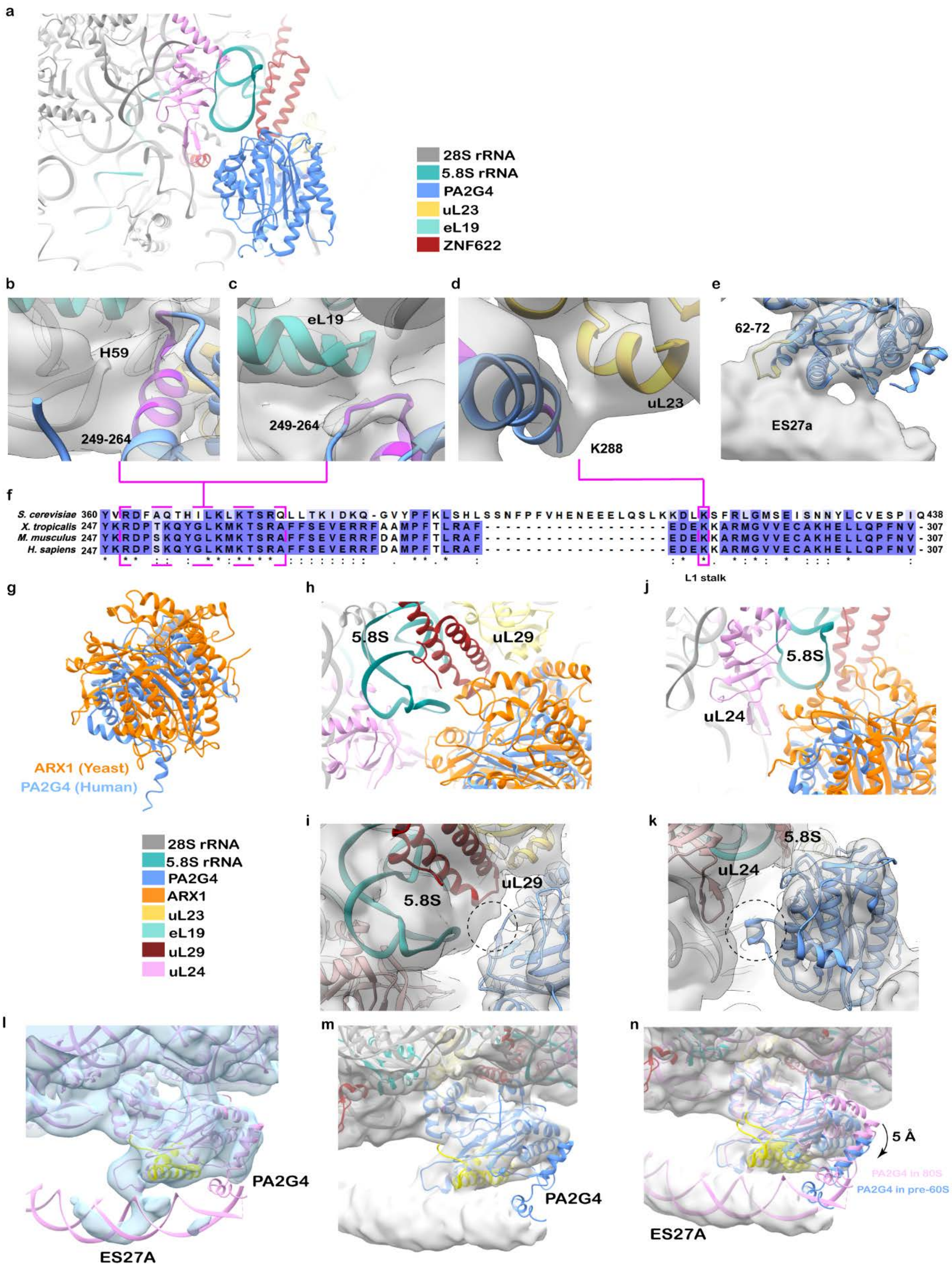
a
28S rRNA
5.8S rRNA
PA2G4
uL23
eL19
ZNF622
b
H59
249-264
c
eL19
249-264
d
uL23
K288
e
62-72
ES27a
f
S. cerevisiae 360 YVRDFAQTHILKLKTSRQLLTKIDKQ-GVYPFKLSHLSSNFPFVHENEEELQSLKKDLKSFRLGMSEISNNYLCVESPIQ 438
X. tropicalis 247 YKRDPTKQYGLKMKTSRAFFSEVERRFAAMPFTLRAF-----------------EDEKKARMGVVECAKHELLQPFNV - 307
M. musculus 247 YKRDPSKQYGLKMKTSRAFFSEVERRFDAMPFTLRAF-----------------EDEKKARMGVVECAKHELLQPFNV - 307
H. sapiens 247 YKRDPSKQYGLKMKTSRAFFSEVERRFDAMPFTLRAF-----------------EDEKKARMGVVECAKHELLQPFNV - 307
L1 stalk
g
ARX1 (Yeast)
PA2G4 (Human)
h
5.8S
uL29
j
5.8S
uL24
28S rRNA
5.8S rRNA
PA2G4
ARX1
uL23
eL19
uL29
uL24
i
5.8S
uL29
k
5.8S
uL24
l
PA2G4
ES27A
m
PA2G4
n
5 Å
PA2G4 in 80S
PA2G4 in pre-60S
ES27A

Supplementary Fig. 9 | Interactions of PA2G4 on the human pre-60S particle.
a, Overview of PA2G4 (PDB 6SXO [7], 2Q8K [8], rigid-body fitting) on the structure of pre-60S particle (state D).
b-d, Conserved interfaces between PA2G4 (magenta) and the ribosomal components (including H59, eL19 and uL23).
e, Residues from 62 to 72 (yellow) containing several positively charged residues probably mediate interactions between PA2G4 and ES27A.
f, Multiple sequence alignment of PA2G4 proteins in conserved regions as shown in (**b**), (**c**). Regions of PA2G4 involved in direct interactions are highlighted in magenta dash-lined squares.
g, Superimposition of PA2G4 and Arx1 (yeast, PDB 5APN) [6]. The structural alignment was performed using the rRNAs as reference.
h-i, Human PA2G4 has no significant contacts with uL29 and the 5.8S rRNA. Superimposition of Arx1 onto the PA2G4-bound pre-60S structure (**h**). The density map of the PA2G4-bound pre-60S structure is superimposed with the model (**i**). As shown, PA2G4 is significantly smaller and is not in close contact with the 5.8S rRNA and uL29.
j-k, Similar as **h-i**, but to highlight that PA2G4 no longer interacts with uL24.
l-n, Comparison of PA2G4 positions in the 80S and pre-60S complexes. PA2G4 in the human 80S-PA2G4 complex (EMD-10344, PDB 6SXO) [7] is shown in cartoon model with superimposed density map (**l**). PA2G4 (blue) in the pre-60S complex of state B (**m**). Superimposition of two models (**n**). As shown, while the 25S/28S rRNAs in the two structures are aligned very well, PA2G4 is seen to have a position shift between two structures. R-proteins and rRNA were used as reference for alignment. Yellow region shows the N-terminal 54 residues of PA2G4 (isoform1).

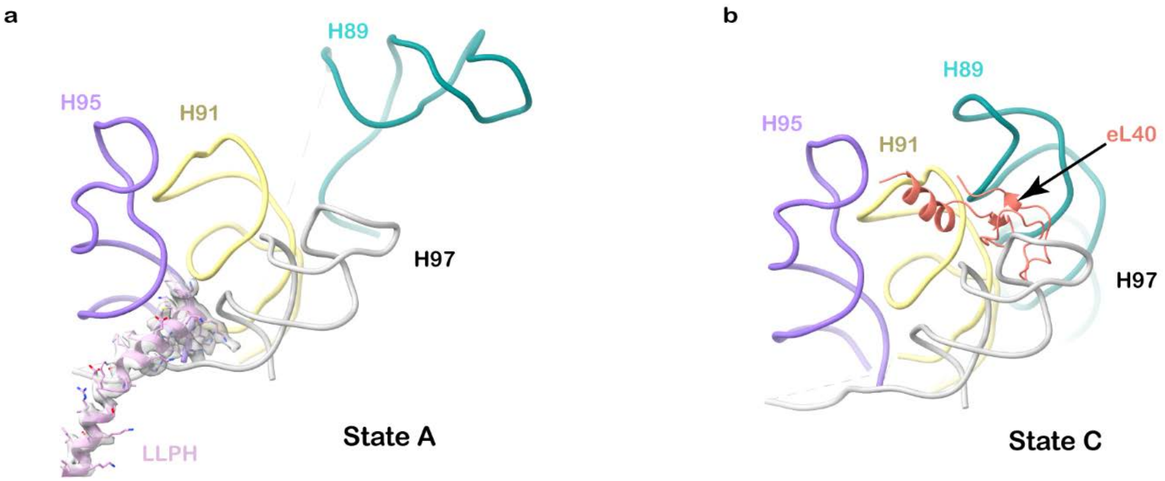

Supplementary Fig. 10 | Structures of LLPH in the human pre-60S particles.
a, LLPH binds to the cleft between H95 and H91 in state A.
b, The binding of eL40 is coupled with the maturation of H89 in state C.

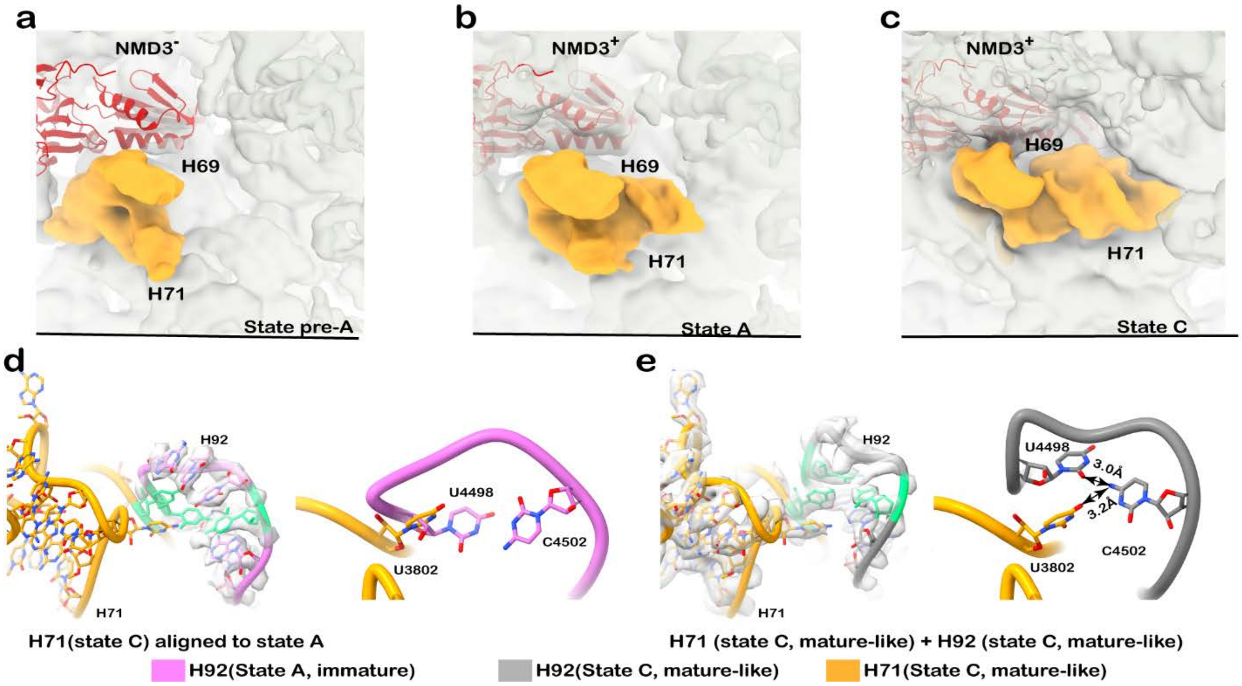

Supplementary Fig. 11 | Progressive maturation of H69-71 in pre-60S structures.
a-c, Cryo-EM maps of the state pre-A, A and C are shown in transparent surface representation, with segmented density of H69-H71 highlighted in gold. The model of NMD3 is also shown. NMD3$^-$ indicates that NMD3 is not stably bound in state pre-A.
d, The tripartite interaction is not formed in state A. Superimposition of H71 (mature-like, state C) onto H92 (state A). The 28S rRNA was used as reference for alignment. As shown, the immature U4494 and C4502 of H92 are in different positions compared to those in state C, and U4498 is particularly in conflict with superimposed U3802 of H71 in state C.
e, Conserved triple base interactions in H71 (U3802) and H92 (U4498, C4502) formed in state C.

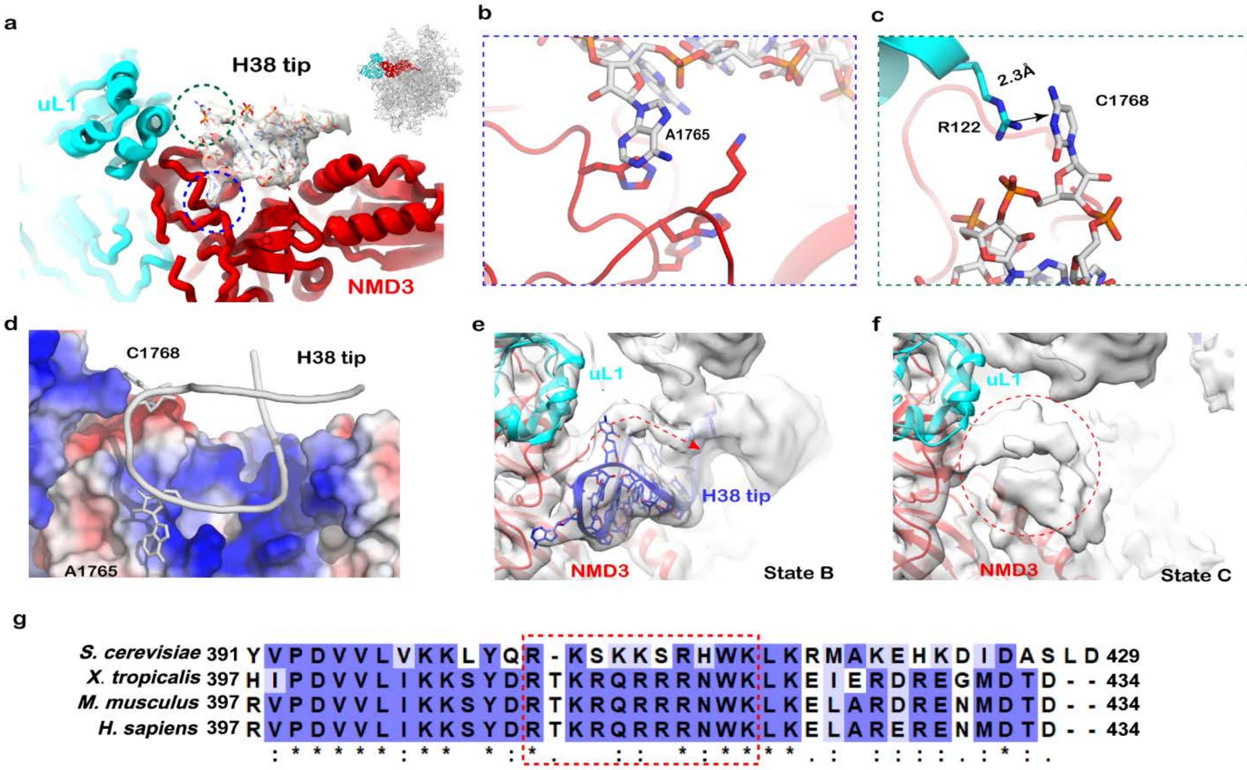

Supplementary Fig. 12 | immature H38 in pre-60S structures.

a, Overall view of the tip of H38 modelled in state B.

b, Structural interface between the tip of H38 and NMD3, highlighting the flipping of A1765 to the OB domain of NMD3.

c, The base of C1768 in H38 points to uL1 and interacts with R122 of uL1.

d, The tip of H38 is stabilized via a highly positively charged binding site formed from NMD3 and uL1. The electrostatic surface potential was generated using PYMOL.

e, Local densities of the tip of H38 and NMD3-CTD in state B. The red dashed arrow shows the path of NMD3-CTD, with a stretch of sequences inserted into the major groove of H38.

f, The same region as in (j), for state C. The red dashed circle shows smear density of NMD3-CTD.

g, Sequence alignment of NMD3 in the region of the major groove insertion motif (red dash-lined square).

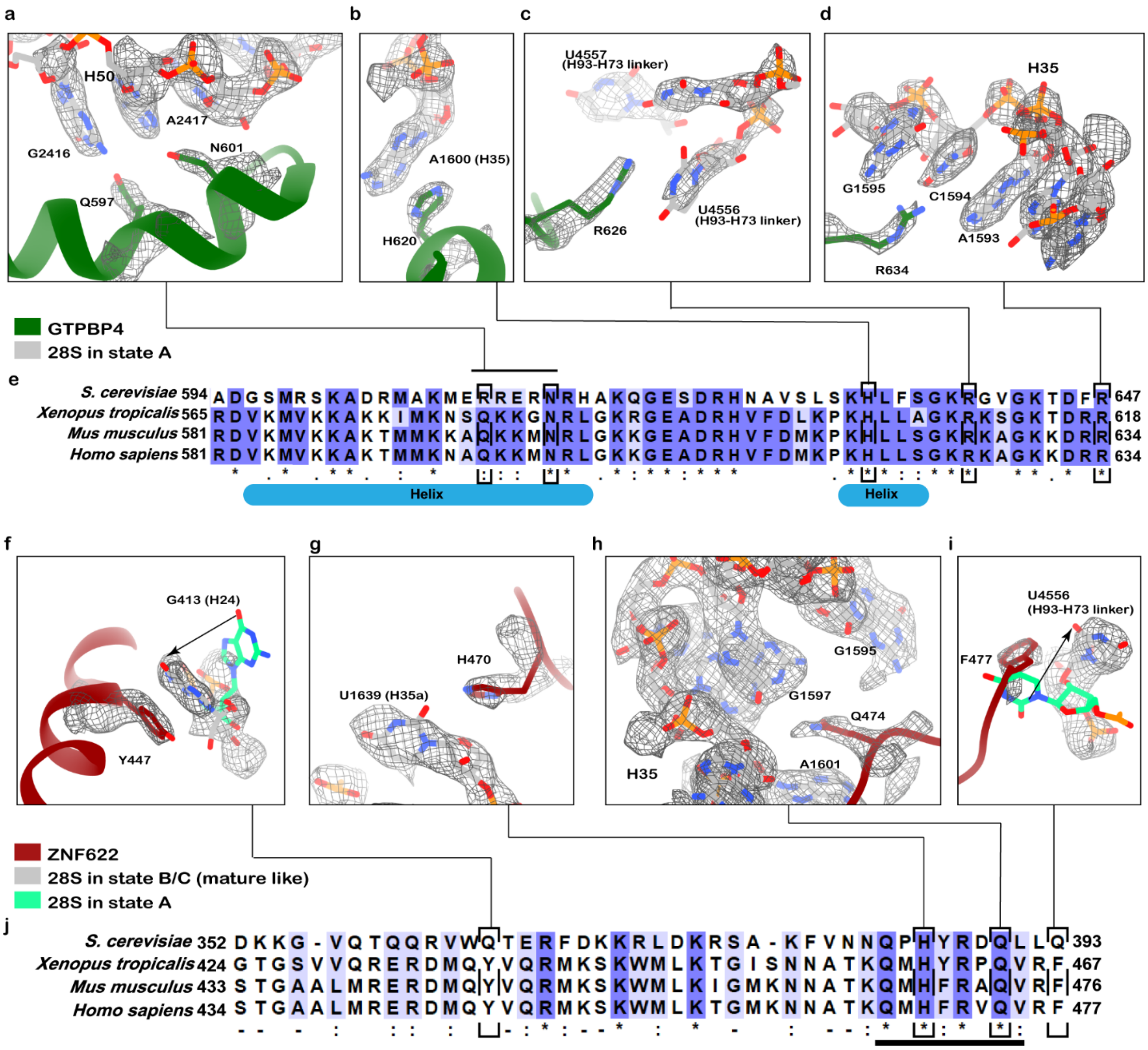

Supplementary Fig. 13 | Interactions of GTPBP4 and ZNF622 tails with the tunnel wall.

a-d, Details of interactions between GTPBP4 and the 28S rRNA in the tunnel. Q597 and N601 interact with bases of G2416 and A2417 from H50 (**a**); H620 stacks with the base of A1600 from H35 (**b**); R626 contacts with the base of U4556 from H73-H93 linker (**c**); R634 interacts with the of C1594 from H35. The atomic models are displayed with superimposed local densities (mesh representation).

e, Multiple sequence alignment of the C-terminal region of GTPBP4. Highly conserved residues mentioned in (**a-d**) are labelled.

f-i, Details of interactions between ZNF622 with the 28S rRNA in tunnel. Y447 stacks with the base of G413 from H24, which leads to the base flip of G413 from state A (green) to state B/C (grey, mature like) (**f**); H470 interacts with the base of U1639 from H35a (**g**); Q474 interacts H35 (**h**). As shown, F477 of ZNF622 from state B is incompatible with immature conformation of U4556 (green) in state A (**i**).

j, Multiple sequence alignment of the C-terminal region of ZNF622. Residues mentioned in f-i are labelled. The conserved Q-X-H-F/Y-R-X-Q motif is shown as black line.

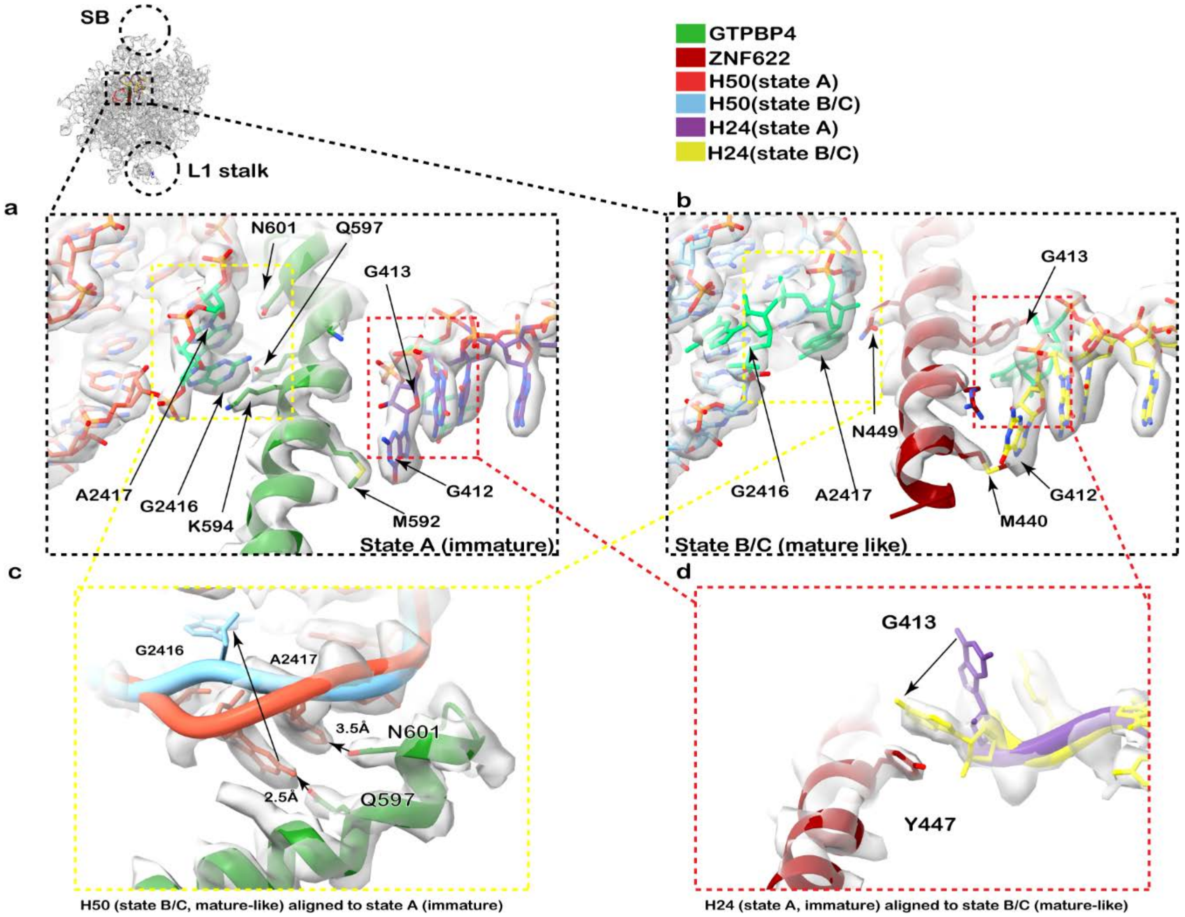

Supplementary Fig. 14 | Interactions of GTPBP4 and ZNF622 near the tunnel exit.

a, Interactions of GTPBP4 with H24 and H50 near the tunnel exit in state A.

b, Interactions of ZNF622 with H24 and H50 in state B/C (mature-like). Density are zoned by model of H50, H24 and two factors in distinct states respectively. Changed bases during maturation are labelled via green

c, Base flip of G2416 from state A (red) to state B/C (light blue, mature-like). Model of H50 in state B/C is aligned to state A, highlighting the polar interactions of GTPBP4 with H50: between Q597 and G2416; between N601 and A2417.

d, Base flip of G413 from state A (purple) to state B/C (yellow, mature-like). Model of H24 in state A is aligned to state B/C, highlighting the stacking interaction between Y447 of ZNF622 and G413 of H24.

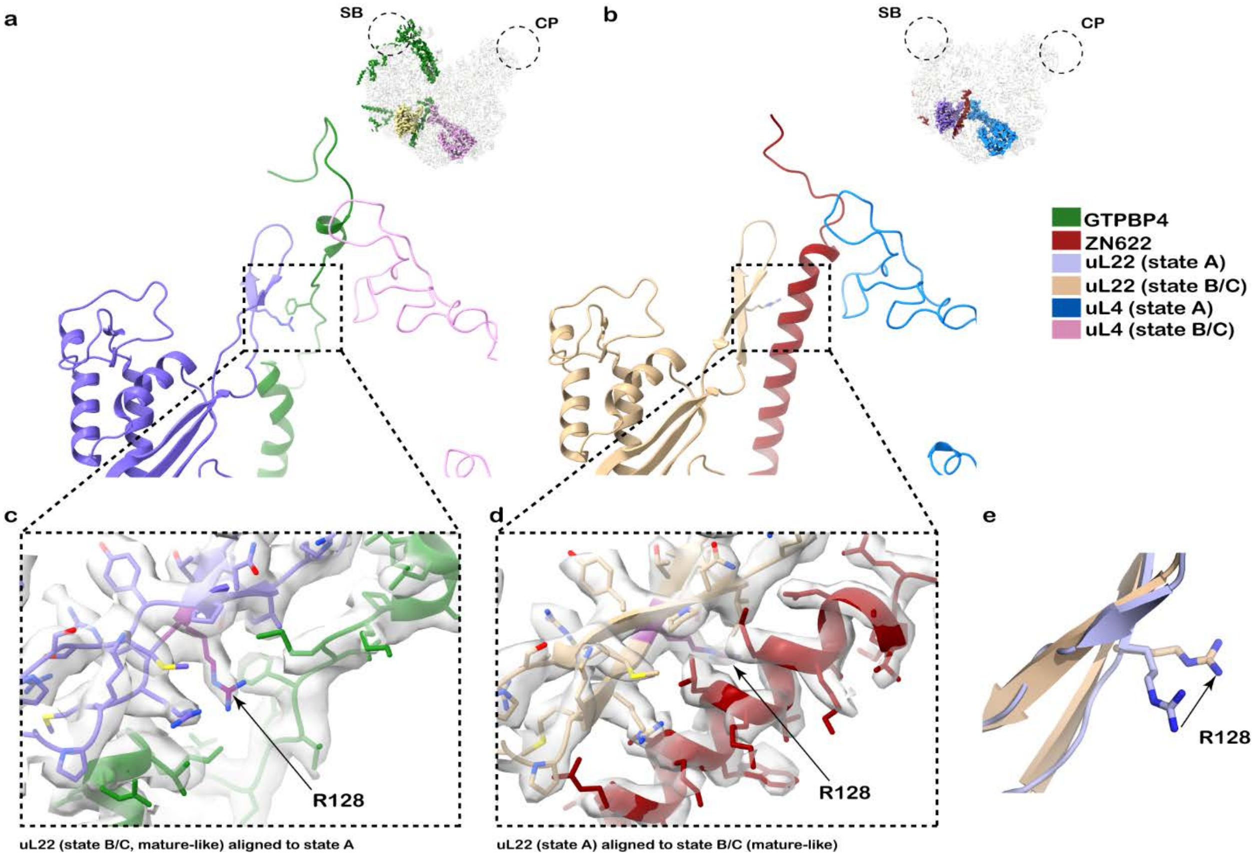

Supplementary Fig. 15 | Side chain reorientation from state A to state B/C of uL22

a-b, Overview of uL22, uL4 and GTPBP4/ZNF622 in the tunnel.

c-d, Zoom-in views on uL22 in GTPBP4-bound (g) and ZNF622-bound states (h).

e, Side-chain rotation of R128 of uL22 from state A (khaki) to state B/C (mature like, light blue).

Supplementary Table 1 | Cryo-EM data collection, refinement and validation statistics

	#1 State pre-A (EMD-0964) (PDB 6LSS	#2 State A (EMD-0978) (PDB 6LU8)	#3 State B (EMD-0963) (PDB 6LSR)	#4 State C (EMD-0948) (PDB 6LQM)
**Data collection and processing**				
Microscope	Titan Krios	Titan Krios	Titan Krios	Titan Krios
Camera	K2	K2	K2	K2
Magnification	130000	130000	130000	130000
Voltage (kV)	300	300	300	300
Electron exposure (e–/Å^2)	64	64	64	64
Electron rate (e–/Å^2/s)	8	8	8	8
Number of frames collected	32	32	32	32
Energy filter slit width (eV)	20	20	20	20
Automation software	SerialEM	SerialEM	SerialEM	SerialEM
Micrographs (no.)	12711	12711	12711	12711
Defocus range (μm)	-1.2— -1.8	-1.2— -1.8	-1.2— -1.8	-1.2— -1.8
Pixel size (Å)	0.529	0.529	0.529	0.529
Symmetry imposed	C1	C1	C1	C1
Initial particle images (no.)	541571<br>101101	541571<br>101101	541571<br>101101	541571<br>101101
Final particle images (no.)	10277	21489	18819	21707
AccuracyRotations	0.4710	0.3400	0.3430	0.3290
AccuracyTranslationsAngst	0.4000	0.3174	0.3174	0.3174
Map resolution (Å)	3.2	3.1	3.1	3.0
FSC threshold	0.143	0.143	0.143	0.143
Map resolution range (Å)	3-6	3-6	3-6	3-6
Map sharpening B factor (Å^2)	-65	-67	-67	-55
**Refinement**				
Initial model used (PDB code)	6EK0, 6N8K	6EK0, 5AJ0, 6N8K	6EK0, 5AJ0, 2Q8K, 6RZZ	6EK0, 5AJ0
Refinement package	phenix.real space	phenix.real space	phenix.real space	phenix.real space
Model map CC				
CC (mask)	0.80	0.81	0.80	0.81
CC (box)	0.78	0.79	0.78	0.80
CC (peaks)	0.71	0.72	0.70	0.74
CC (volumn)	0.80	0.81	0.80	0.82
Model composition				
Non-hydrogen atoms	142452	149096	143598	140715
Protein residues	7416	7883	7587	7116
Nucleotide	3793	3962	3793	3836
Ligands	276	278	274	278
B factors (Å^2)				
Protein	12.25	13.12	13.10	13.27
Nucleotide	25.50	28.29	33.01	24.97
Ligand	14.53	11.74	8.88	11.90
R.m.s. deviations				
Bond lengths (Å)	0.008	0.009	0.011	0.014
Bond angles (°)	1.029	1.357	1.142	1.228
Cβ outliers	0.03	0.04	0.00	0.02
CaBLAM outliers	4.15	4.62	4.27	4.18
Validation				
MolProbity score	1.76	1.59	1.77	1.57

Clashscore	5.24	2.75	4.34	3.54
Poor rotamers (%)	0.73	1.20	1.22	0.50
Ramachandran plot				
Favored (%)	92.16	92.60	92.00	92.36
Allowed (%)	7.74	7.25	7.84	7.47
Disallowed (%)	0.10	0.16	0.16	0.17

Supplementary Table 2 | Mass spectrometry analysis of purified NMD3-particles.

AC	Score	Coverage	No. of peptide	Description
sp|Q9NVN8|GNL3L_HUMAN	6.7	0.797	226	Guanine nucleotide-binding protein-like 3-like protein OS=Homo sapiens GN=GNL3L PE=1 SV=1
sp|Q9NU22|MDN1_HUMAN	6.612	0.593	327	Midasin OS=Homo sapiens GN=MDN1 PE=1 SV=2
sp|P42285|SK2L2_HUMAN	6.535	0.618	89	Superkiller viralicidic activity 2-like 2 OS=Homo sapiens GN=SKIV2L2 PE=1 SV=3
sp|Q8NB90|SPAT5_HUMAN	6.361	0.688	55	Spermatogenesis-associated protein 5 OS=Homo sapiens GN=SPATA5 PE=1 SV=3
sp|P05386|RLA1_HUMAN	6.346	0.991	27	60S acidic ribosomal protein P1 OS=Homo sapiens GN=RPLP1 PE=1 SV=1
sp|Q9UKD2|MRT4_HUMAN	6.278	0.674	76	mRNA turnover protein 4 homolog OS=Homo sapiens GN=MRTO4 PE=1 SV=2
sp|Q9UNI6|DUS12_HUMAN	6.252	0.874	33	Dual specificity protein phosphatase 12 OS=Homo sapiens GN=DUSP12 PE=1 SV=1
sp|P56537|IF6_HUMAN	6.247	0.857	84	Eukaryotic translation initiation factor 6 OS=Homo sapiens GN=EIF6 PE=1 SV=1
sp|P30050|RL12_HUMAN	5.796	0.927	85	60S ribosomal protein L12 OS=Homo sapiens GN=RPL12 PE=1 SV=1
sp|Q13823|NOG2_HUMAN	5.791	0.423	30	Nucleolar GTP-binding protein 2 OS=Homo sapiens GN=GNL2 PE=1 SV=1
sp|Q969S3|ZN622_HUMAN	5.778	0.503	55	Zinc finger protein 622 OS=Homo sapiens GN=ZNF622 PE=1 SV=1
sp|Q9UQ80|PA2G4_HUMAN	5.607	0.728	60	Proliferation-associated protein 2G4 OS=Homo sapiens GN=PA2G4 PE=1 SV=3
sp|Q96D46|NMD3_HUMAN	5.494	0.982	707	60S ribosomal export protein NMD3 OS=Homo sapiens GN=NMD3 PE=1 SV=1
sp|Q9BRT6|LLPH_HUMAN	5.493	0.504	47	Protein LLP homolog OS=Homo sapiens GN=LLPH PE=1 SV=1
sp|P05387|RLA2_HUMAN	5.279	1	75	60S acidic ribosomal protein P2 OS=Homo sapiens GN=RPLP2 PE=1 SV=1
sp|Q9BZE4|NOG1_HUMAN	5.048	0.763	281	Nucleolar GTP-binding protein 1 OS=Homo sapiens GN=GTPBP4 PE=1 SV=3
sp|Q8IY81|SPB1_HUMAN	4.77	0.294	19	pre-rRNA processing protein FTSJ3 OS=Homo sapiens GN=FTSJ3 PE=1 SV=2
sp|Q9H089|LSG1_HUMAN	4.723	0.745	68	Large subunit GTPase 1 homolog OS=Homo sapiens GN=LSG1 PE=1 SV=2
sp|P05388|RLA0_HUMAN	4.714	0.782	104	60S acidic ribosomal protein P0 OS=Homo sapiens GN=RPLP0 PE=1 SV=1
sp|O95478|NSA2_HUMAN	4.652	0.535	23	Ribosome biogenesis protein NSA2 homolog OS=Homo sapiens GN=NSA2 PE=1 SV=1
sp|Q9NX58|LYAR_HUMAN	4.517	0.332	11	Cell growth-regulating nucleolar protein OS=Homo sapiens GN=LYAR PE=1 SV=2
sp|Q8TDN6|BRX1_HUMAN	4.505	0.499	12	Ribosome biogenesis protein BRX1 homolog OS=Homo sapiens GN=BRIX1 PE=1 SV=2
sp|Q9Y4W2|LAS1L_HUMAN	4.311	0.168	9	Ribosomal biogenesis protein LAS1L OS=Homo sapiens GN=LAS1L PE=1 SV=2
sp|Q9NZM5|GSCR2_HUMAN	4.25	0.462	26	Glioma tumor suppressor candidate region gene 2 protein OS=Homo sapiens GN=GLTSCR2 PE=1 SV=2
sp|Q15050|RRS1_HUMAN	4.126	0.425	17	Ribosome biogenesis regulatory protein homolog OS=Homo sapiens GN=RRS1 PE=1 SV=2
sp|O00488|ZN593_HUMAN	3.941	0.784	63	Zinc finger protein 593 OS=Homo sapiens GN=ZNF593 PE=1 SV=2
sp|Q5F1R6|DJC21_HUMAN	3.869	0.1	4	DnaJ homolog subfamily C member 21 OS=Homo sapiens GN=DNAJC21 PE=1 SV=2
sp|Q9H0D6|XRN2_HUMAN	3.809	0.261	18	5'-3' exoribonuclease 2 OS=Homo sapiens GN=XRN2 PE=1 SV=1
sp|Q9NVP1|DDX18_HUMAN	3.784	0.2	10	ATP-dependent RNA helicase DDX18 OS=Homo sapiens GN=DDX18 PE=1 SV=2
sp|Q8WTT2|NOC3L_HUMAN	3.686	0.099	5	Nucleolar complex protein 3 homolog OS=Homo sapiens GN=NOC3L PE=1 SV=1
tr|J3KTA4|J3KTA4_HUMAN	3.533	0.542	39	Probable ATP-dependent RNA helicase DDX5 OS=Homo sapiens GN=DDX5 PE=1 SV=1
sp|Q9UHA3|RLP24_HUMAN	3.531	0.656	49	Probable ribosome biogenesis protein RLP24 OS=Homo sapiens GN=RSL24D1 PE=1 SV=1
tr|H0Y9X1|H0Y9X1_HUMAN	3.478	0.471	66	Translation machinery-associated protein 16 (Fragment) OS=Homo sapiens GN=TMA16 PE=1 SV=1
sp|Q9GZL7|WDR12_HUMAN	3.332	0.196	6	Ribosome biogenesis protein WDR12 OS=Homo sapiens GN=WDR12 PE=1 SV=2
sp|Q9Y3C1|NOP16_HUMAN	3.303	0.517	15	Nucleolar protein 16 OS=Homo sapiens GN=NOP16 PE=1 SV=2
sp|Q96P11|NSUN5_HUMAN	3.131	0.117	3	Probable 28S rRNA (cytosine-C(5))-methyltransferase OS=Homo sapiens GN=NSUN5 PE=1 SV=2
sp|Q14137|BOP1_HUMAN	3.053	0.206	15	Ribosome biogenesis protein BOP1 OS=Homo sapiens GN=BOP1 PE=1 SV=2
sp|Q9NVX2|NLE1_HUMAN	3.023	0.443	16	Notchless protein homolog 1 OS=Homo sapiens GN=NLE1 PE=1 SV=4

sp|P46087|NOP2_HUMAN	3.022	0.11	7	Probable 28S rRNA (cytosine(4447)-C(5))-methyltransferase OS=Homo sapiens GN=NOP2 PE=1 SV=2
tr|B5MCF9|B5MCF9_HUMAN	2.769	0.483	31	Pescadillo homolog OS=Homo sapiens GN=PES1 PE=1 SV=1
sp|Q8TDD1|DDX54_HUMAN	2.598	0.185	11	ATP-dependent RNA helicase DDX54 OS=Homo sapiens GN=DDX54 PE=1 SV=2
sp|Q9H7B2|RPF2_HUMAN	2.597	0.425	12	Ribosome production factor 2 homolog OS=Homo sapiens GN=RPF2 PE=1 SV=2
tr|G3V529|G3V529_HUMAN	2.365	0.342	23	ATP-dependent RNA helicase DDX24 OS=Homo sapiens GN=DDX24 PE=1 SV=1
tr|U3KQC1|U3KQC1_HUMAN	2.363	0.236	6	WD repeat-containing protein 18 (Fragment) OS=Homo sapiens GN=WDR18 PE=1 SV=1
sp|Q9Y3T9|NOC2L_HUMAN	2.32	0.067	5	Nucleolar complex protein 2 homolog OS=Homo sapiens GN=NOC2L PE=1 SV=4
tr|C9JFV4|C9JFV4_HUMAN	2.27	0.175	14	Proline-, glutamic acid- and leucine-rich protein 1 OS=Homo sapiens GN=PELP1 PE=1 SV=2
tr|A0A0A6YYI3|A0A0A6YYI3_HUMAN	2.24	0.181	5	Protein PPAN-P2RY11 OS=Homo sapiens GN=PPAN-P2RY11 PE=4 SV=1
tr|H7C2Q8|H7C2Q8_HUMAN	1.922	0.266	9	EBNA1 binding protein 2, isoform CRA_d OS=Homo sapiens GN=EBNA1BP2 PE=1 SV=1

Supplementary References

1 Natchiar, S. K., Myasnikov, A. G., Kratzat, H., Hazemann, I. & Klaholz, B. P. Visualization of chemical modifications in the human 80S ribosome structure. *Nature* **551**, 472-477, doi:10.1038/nature24482 (2017).

2 Behrmann, E. *et al.* Structural snapshots of actively translating human ribosomes. *Cell* **161**, 845-857, doi:10.1016/j.cell.2015.03.052 (2015).

3 Zhou, Y., Musalgaonkar, S., Johnson, A. W. & Taylor, D. W. Tightly-orchestrated rearrangements govern catalytic center assembly of the ribosome. *Nat Commun* **10**, 958, doi:10.1038/s41467-019-08880-0 (2019).

4 Wu, S. *et al.* Diverse roles of assembly factors revealed by structures of late nuclear pre-60S ribosomes. *Nature* **534**, 133-137, doi:10.1038/nature17942 (2016).

5 Kater, L. *et al.* Visualizing the Assembly Pathway of Nucleolar Pre-60S Ribosomes. *Cell* **171**, 1599-1610 e1514, doi:10.1016/j.cell.2017.11.039 (2017).

6 Greber, B. J. *et al.* Insertion of the Biogenesis Factor Rei1 Probes the Ribosomal Tunnel during 60S Maturation. *Cell* **164**, 91-102, doi:10.1016/j.cell.2015.11.027 (2016).

7 Wild, K. *et al.* MetAP-like Ebp1 occupies the human ribosomal tunnel exit and recruits flexible rRNA expansion segments. *Nat Commun* **11**, 776, doi:10.1038/s41467-020-14603-7 (2020).

8 Kowalinski, E. *et al.* The crystal structure of Ebp1 reveals a methionine aminopeptidase fold as binding platform for multiple interactions. *FEBS Lett* **581**, 4450-4454, doi:10.1016/j.febslet.2007.08.024 (2007).
