# Supplementary material for: Structural snapshots of human pre-60S ribosomal particles before and after nuclear export
Source: Nat Commun. 2020 Jul 15;11:3542. doi: 10.1038/s41467-020-17237-x (PMC7363849; doi:10.1038/s41467-020-17237-x)
Supplement: Supplementary file 3 — Reporting Summary [file 41467_2020_17237_MOESM3_ESM.pdf]

nature research

Corresponding author(s): Ning Gao

Last updated by author(s): Jun 19, 2020

# Reporting Summary

Nature Research wishes to improve the reproducibility of the work that we publish. This form provides structure for consistency and transparency in reporting. For further information on Nature Research policies, see our Editorial Policies and the Editorial Policy Checklist.

## Statistics

For all statistical analyses, confirm that the following items are present in the figure legend, table legend, main text, or Methods section.

| n/a | Confirmed | |
|---|---|---|
| [ ] | [x] | The exact sample size (*n*) for each experimental group/condition, given as a discrete number and unit of measurement |
| [ ] | [x] | A statement on whether measurements were taken from distinct samples or whether the same sample was measured repeatedly |
| [x] | [ ] | The statistical test(s) used AND whether they are one- or two-sided<br>*Only common tests should be described solely by name; describe more complex techniques in the Methods section.* |
| [x] | [ ] | A description of all covariates tested |
| [x] | [ ] | A description of any assumptions or corrections, such as tests of normality and adjustment for multiple comparisons |
| [x] | [ ] | A full description of the statistical parameters including central tendency (e.g. means) or other basic estimates (e.g. regression coefficient) AND variation (e.g. standard deviation) or associated estimates of uncertainty (e.g. confidence intervals) |
| [x] | [ ] | For null hypothesis testing, the test statistic (e.g. *F*, *t*, *r*) with confidence intervals, effect sizes, degrees of freedom and *P* value noted<br>*Give P values as exact values whenever suitable.* |
| [x] | [ ] | For Bayesian analysis, information on the choice of priors and Markov chain Monte Carlo settings |
| [x] | [ ] | For hierarchical and complex designs, identification of the appropriate level for tests and full reporting of outcomes |
| [x] | [ ] | Estimates of effect sizes (e.g. Cohen's *d*, Pearson's *r*), indicating how they were calculated |

*Our web collection on statistics for biologists contains articles on many of the points above.*

## Software and code

Policy information about availability of computer code

| | |
|---|---|
| Data collection | SerialEM |
| Data analysis | Relion-3.0, Relion-3.1-Beta, MotionCorr 2.1, Gctf v1.0.6, Spider-23.02, Chimera64-1.11.2, EMAN2.2, coot-0.8.7, Chimera 1.11.2, ChimeraX 0.9.3, Pymol 2.0.6, Phenix-dev-3689, Phenix-1.1.4.3260, SwissModel, i-TASSER, T-coffee, BioEdit, |

For manuscripts utilizing custom algorithms or software that are central to the research but not yet described in published literature, software must be made available to editors and reviewers. We strongly encourage code deposition in a community repository (e.g. GitHub). See the Nature Research guidelines for submitting code & software for further information.

## Data

Policy information about availability of data

All manuscripts must include a data availability statement. This statement should provide the following information, where applicable:

- Accession codes, unique identifiers, or web links for publicly available datasets
- A list of figures that have associated raw data
- A description of any restrictions on data availability

Cryo-EM density maps have been deposited in the EM Data Bank under the accession codes EMD-0964, EMD-0978, EMD-0963, EMD-0948 for state pre-A to state C. Atomic models of the four states have been deposited in Protein Data Bank under 6LSS, 6LU8, 6LSR and 6LQM.



April 2020

# Field-specific reporting

Please select the one below that is the best fit for your research. If you are not sure, read the appropriate sections before making your selection.

[x] Life sciences  [ ] Behavioural & social sciences  [ ] Ecological, evolutionary & environmental sciences

For a reference copy of the document with all sections, see nature.com/documents/nr-reporting-summary-flat.pdf

# Life sciences study design

All studies must disclose on these points even when the disclosure is negative.

| | |
|---|---|
| Sample size | No statistical methods were applied to pre-determine sample sizes |
| Data exclusions | No data were excluded from the analysis other than the 2D and 3D classification during image processing |
| Replication | All biochemical and biological experiments were successfully and reliably reproduced. |
| Randomization | No randomization was required for the reported experiments |
| Blinding | Blinding was not required for the reported experiments |

# Reporting for specific materials, systems and methods

We require information from authors about some types of materials, experimental systems and methods used in many studies. Here, indicate whether each material, system or method listed is relevant to your study. If you are not sure if a list item applies to your research, read the appropriate section before selecting a response.

## Materials & experimental systems

| n/a | Involved in the study | |
|---|---|---|
| [x] | [ ] | Antibodies |
| [ ] | [x] | Eukaryotic cell lines |
| [x] | [ ] | Palaeontology and archaeology |
| [x] | [ ] | Animals and other organisms |
| [x] | [ ] | Human research participants |
| [x] | [ ] | Clinical data |
| [x] | [ ] | Dual use research of concern |

## Methods

| n/a | Involved in the study | |
|---|---|---|
| [x] | [ ] | ChIP-seq |
| [x] | [ ] | Flow cytometry |
| [x] | [ ] | MRI-based neuroimaging |

# Eukaryotic cell lines

Policy information about cell lines

| | |
|---|---|
| Cell line source(s) | HEK293FT |
| Authentication | Cells were not authenticated |
| Mycoplasma contamination | The cell line used in this work was not tested for mycoplasma contamination. |
| Commonly misidentified lines (See ICLAC register) | No cell lines used throughout the work are listed in the ICLAC database |
