# Supplementary material for: Structural snapshots of human pre-60S ribosomal particles before and after nuclear export
Source: Nat Commun. 2020 Jul 15;11:3542. doi: 10.1038/s41467-020-17237-x (PMC7363849; doi:10.1038/s41467-020-17237-x)
Supplement: Supplementary file 4 — Description of Additional Supplementary Files [file 41467_2020_17237_MOESM4_ESM.docx]

**Description of Additional Supplementary Files**

File name: Supplementary Movie 1

Description: Structures of human pre-60S particles isolated via C-terminally tagged NMD3.

Cryo-EM maps of state pre-A to C (gaussian filtered with sDev of 1.058 Å in ChimeraX) are displayed sequentially. Individual assembly factors, H38, H89, L1 stalk, eL24, eL40, uL1 and uL16 are color-coded.

File name: Supplementary Movie 2

Description: Location of Protein X in the map of state pre-A.

Cryo-EM map of state pre-A (gaussian filtered with sDev of 1.058 Å in ChimeraX) is shown with TMA16, GTPBP4, uL6, H89, H91, H97, H42 and H41-H42 linker color-coded. Segmented map of protein X is filtered with sDev of 2.0 Å to facilitate the illustration.

File name: Supplementary Movie 3

Description: Conformational changes of H89 from state A to state C.

The animation shows a continuous morphing of H89 from state A, state B to state C. Models of uL16 and eL40 are from state C. The model of NMD3 is from state B (NMD3-NTD is derived from a previous structure 6RZZ).

File name: Supplementary Movie 4

Description: Conformational change of H38 from state A/B to state C.

The animation shows a morphing of H38 from stateA/B to state C. The position of NMD3 is from state B (NMD3-NTD is derived from a previous structure 6RZZ). Protein uL16 is from state C.
