# Supplementary material for: Structural snapshots of human pre-60S ribosomal particles before and after nuclear export
Source: Nat Commun. 2020 Jul 15;11:3542. doi: 10.1038/s41467-020-17237-x (PMC7363849; doi:10.1038/s41467-020-17237-x)
Supplement: Supplementary file 2 — Peer Review File [file 41467_2020_17237_MOESM2_ESM.pdf]

Editorial Note: This manuscript has been previously reviewed at another journal that is not operating a transparent peer review scheme. This document only contains reviewer comments and rebuttal letters for versions considered at Nature Communications.
